# Regional variation in growth and survival responses to atmospheric nitrogen and sulfur deposition for 140 tree species across the United States

**DOI:** 10.3389/ffgc.2024.1426644

**Published:** 2024-11-11

**Authors:** Rebecca M. Dalton, Jesse N. Miller, Tara Greaver, Robert D. Sabo, Kemen G. Austin, Jennifer N. Phelan, R. Quinn Thomas, Christopher M. Clark

**Affiliations:** 1 U.S. Environmental Protection Agency, Office of Research and Development, Center for Public Health and Environmental Assessment, Research Triangle Park, Durham, NC, United States; 2 U.S. Environmental Protection Agency, Office of Research and Development, Center for Public Health and Environmental Assessment, Washington, DC, United States; 3 Research Triangle Institute (RTI) International, Research Triangle Park, Durham, NC, United States; 4 Department of Forest Resources and Environmental Conservation, Virginia Tech, Cheatham Hall, Blacksburg, VA, United States; 5 Department of Biological Sciences, Virginia Tech, Derring Hall, Blacksburg, VA, United States

**Keywords:** climate, critical load, forest inventory analysis (FIA), nitrogen deposition, sulfur deposition, tree growth, tree survival, vulnerability

## Abstract

Atmospheric deposition of nitrogen (N) and sulfur (S) alter tree demographic processes via changes in nutrient pools, soil acidification, and biotic interactions. Previous work established tree growth and survival response to atmospheric N and S deposition in the conterminous United States (CONUS) data by species; however, it was not possible to evaluate regional variation in response using that approach. In this study, we develop species- and region-specific relationships for growth and survival responses to N and S deposition for roughly 140 species within spatially demarcated regions of the U.S. We calculated responses to N and S deposition separately for 11 United States Forest Service (USFS) Divisions resulting in a total of 241 and 268 species × Division combinations for growth and survival, respectively. We then assigned these relationships into broad categories of vulnerability and used ordinal logistic regressions to explore the covariates associated with vulnerability in growth and survival to N and S deposition. As with earlier studies, we found growth and survival responses to air pollution differed by species; but new to this study, we found 45%−70% of species responses also varied spatially across regions. The regional variation in species responses was not simply related to atmospheric N and S deposition, but was also associated with regional effects from precipitation, soil pH, mycorrhizal association, and deciduousness. A large amount of the variance remained unexplained (total variation explained ranged from 6.8%−13.8%), suggesting that these or additional factors may operate at finer spatial scales. Taken together, our results demonstrate that regional variation in tree species’ response has significant implications for setting critical load targets, as critical loads can now be tailored for specific species at management-relevant scales.

## 1 Introduction

Forests cover about one third of the land surface (Keenan et al., 2015) and are essential components of the earth’s energy, water, carbon, and nutrient cycles. A near ubiquitous and chronic stressor to forest health in most industrial countries, and an emerging threat in many developing countries, is the emissions and subsequent air pollution deposition of nitrogen (N) and sulfur (S) compounds (referenced as N and S) from anthropogenic activities (Galloway et al., 2008; Zhong et al., 2020; Benish et al., 2022). Nitrogen and sulfur deposition, at high enough rates, can acidify (N and S) and eutrophy (N) forests altering tree growth and mortality. Although N and S deposition are declining over much of the eastern United States (U.S.) and other industrialized countries (Lloret and Valiela, 2016; Galloway et al., 2004), both are still elevated far over pre-industrial levels (Galloway et al., 2004) and over many thresholds of known ecological effects (Lynch et al., 2020; Pardo et al., 2011).

Multiple empirical studies conducted in both the U.S. and Europe have demonstrated that specific tree species have varying levels of sensitivity to N and S deposition (Thomas et al., 2010; Clark et al., 2021; Horn et al., 2018; Etzold et al., 2020). Horn et al. (2018) and Thomas et al. (2010) developed species-specific curves that relate N and S deposition to tree growth and survival responses in the U.S. using spatially aggregated data from individuals of the same species across their full population range. Canham and Murphy (2016) and Canham and Murphy (2017) developed similar responses to N for the 50 dominate tree species in the eastern U.S. More recently, machine learning has been used to characterize the associations between N deposition and tree growth and survival at the national-scale (Pavlovic et al., 2023). Regardless of the statistical method employed, these response curves give insight into how tree species respond to changes in N and/or S deposition across their full range (Horn et al., 2018; Thomas et al., 2010; Pavlovic et al., 2023; Canham and Murphy, 2017, 2016). While these large-scale aggregations are an important way to understand the central tendency of the tree species response, it remains unclear whether growth and survival response curves for individual species vary spatially across the U.S., and what co-variates contribute to that geographic variation in response.

There is an emerging body of evidence describing how spatial variation in abiotic environmental factors influence tree response to N and S deposition. Abiotic factors that could alter tree growth and survival responses to N and S include climate (Ibáñez et al., 2018), evapotranspiration and soil water relations (Fan et al., 2022; Gessler et al., 2017), and soil fertility [e.g., base cations, N, carbon (C), C:N ratio, pH] (DesRochers et al., 2007). Soil base cations associated with acidifying deposition [i.e., base cation (BC)/Aluminum (Al), Calcium (Ca)/Al and % base saturation] harmful to trees are well documented, and national-scale estimates of critical loads to N and S deposition are sensitive to soil base cation weathering in the U.S. (McDonnell et al., 2023; McNulty et al., 2007). Growth response is also reported to be lower with elevated potential evapotranspiration, which would incorporate both temperature and precipitation influences of climate (Schulte-Uebbing et al., 2022).

Not only do regional abiotic conditions affect tree growth and survival response to atmospheric N and S deposition, but responses may also vary spatially due to biotic factors relating to tree functional traits. For example, leaf habit is a broad categorization capturing a suite of physiological differences (e.g., deciduous leaves have higher rates of photosynthesis; evergreens have slower growth rates and longer photosynthetic seasons). Specifically, deciduous trees are more likely to allocate increased N to increasing photosynthetic capacity, leading to an increase in growth rate potential with additional N fertilization (Takashima et al., 2004). In addition to deciduousness, another important biotic factor potentially influencing the response of trees to N deposition is symbiosis with mycorrhizae. Associations with mycorrhizal fungi are known to be an advantage to trees in nutrient limited environments; mycorrhizae provide access to N and other nutrients to the tree and the tree provides C to the mycorrhizae (Phillips et al., 2013). The type of mycorrhizal association [e.g., arbuscular mycorrhizal (AM) vs. ectomycorrhizal (EcM)], for a given tree species, is also shown to be an important predictor in tree growth response to N deposition (Thomas et al., 2010; Averill et al., 2018). The mechanisms relating mycorrhizal type to tree growth related to N deposition in the above studies is unclear, it could be that N addition decreases AM root colonization and associated C costs to the trees allowing more C for tree growth, or it may be that because AM fungi do not produce exoenzymes that break down soil organic N, trees with AM-fungi may be more linked to inorganic sources of N including deposition.

**FIGURE 1:**
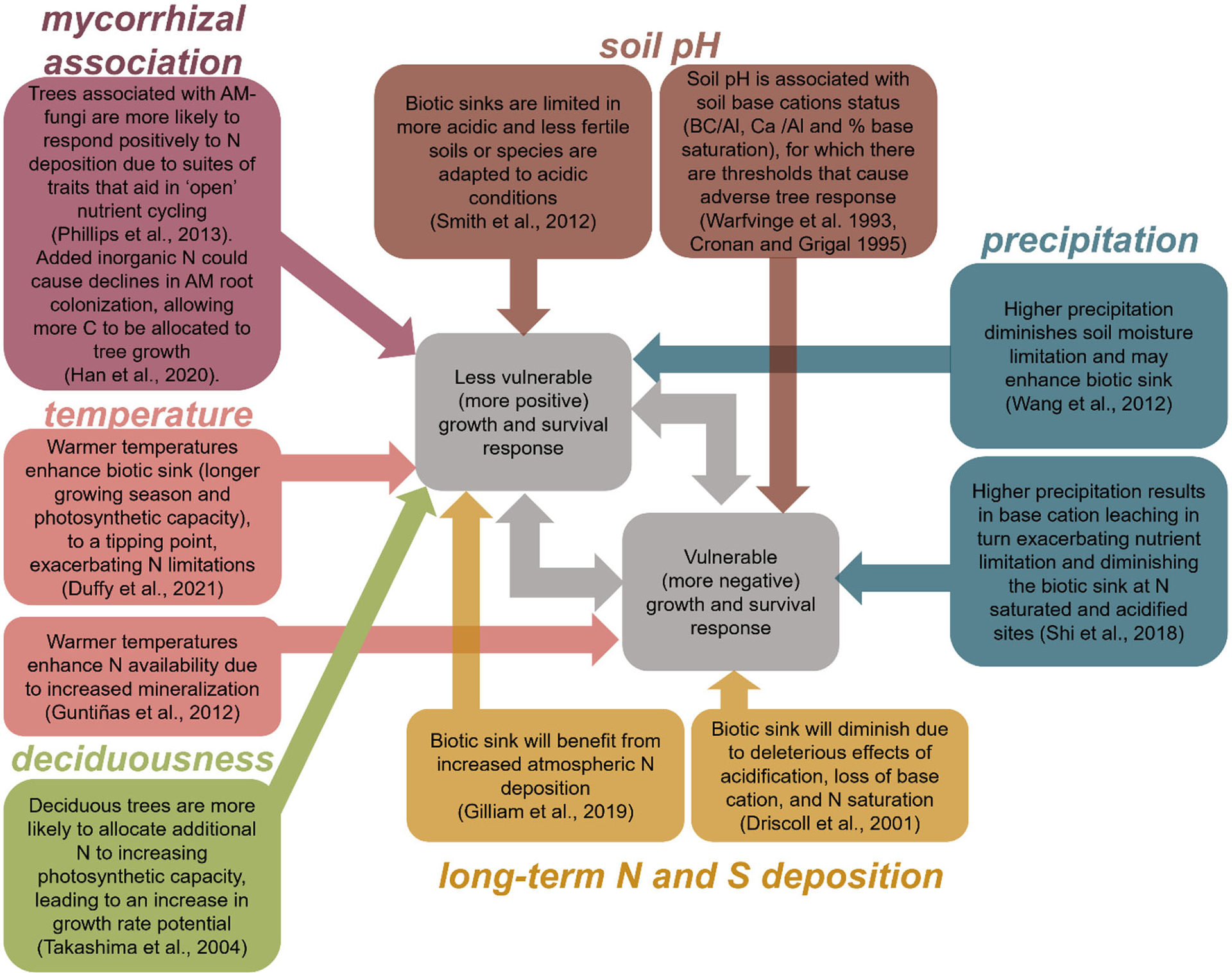
Non-exhaustive diagram of hypothesized associations between climate (temperature, precipitation), soil pH, atmospheric N and S deposition, and tree functional traits (deciduousness and mycorrhizal association) and tree growth/survival responses to increasing N and S deposition. Although we grouped species’ growth and survival responses to N and S deposition into vulnerable (decreasing growth rate or survival probability) and less vulnerable responses (increasing growth rate or survival probability), species’ may exhibit a unimodal or sigmoidal response due to transition of effects from multiple mechanisms, or no response due to multiple mechanisms.

Here, we expanded on the earlier studies of Thomas et al. (2010) and Horn et al. (2018) to establish whether significant regional species-specific differences exist in tree growth rate and probability of survival responses to atmospheric N and S deposition and identify predominant drivers of the regional variation in response. First, we characterized regional variation in tree growth rate and probability of survival responses to N and S deposition. Second, we assessed whether regional variation in these responses was correlated with climate (mean annual temperature and precipitation), soil pH, N, and S deposition, and tree functional traits (mycorrhizal association and deciduousness). Many of these factors have complex and non-linear ecological effects, such that they may increase or decrease vulnerability to atmospheric deposition. For example, low temperatures may exacerbate N limitation, leading to a larger effect from atmospheric N deposition; but higher temperatures may be associated with water-limitation, leading to a weaker effect from atmospheric deposition. We are briefly summarized hypotheses in Figure 1.

## 2 Materials and methods

### 2.1 Tree database

We used a similar dataset and methods as Horn et al. (2018) to derive species-level responses to N and S deposition; however, instead of national-level aggregations of species, we partitioned data into smaller regions (see Section 2.1.1). Tree growth, tree survival, and plot-level basal area data were compiled from the United States Forest Service (USFS) Forest Inventory and Analysis (FIA) program database (accessed on January 24, 2017, FIA phase 2 manual version 6.1:http://www.fia.fs.fed.us/). We used publicly available locations of private, state, and federal lands for this study.True coordinates are roughly within 1 km^2^ of the actual plot location for plots located on public lands. Approximately 20% of plots on private lands are swapped with other private lands that have similar forest characteristics. Studies have found that using public coordinates for spatial data within the 1 km^2^ resolution have negligible impacted results (Gibson et al., 2014). Given the resolution of our environmental variables is rather large (e.g., the finest scale was 4 km × 4 km), and changes between adjacent grid cells are likely small, we do not anticipate a large impact of using public coordinates. That said, we are mindful of these limitations when examining results from this study. We estimated aboveground tree biomass from tree diameter measurements related to allometric relationships (Jenkins et al., 2003) and then multiplied by 0.5 to estimate aboveground C. We are aware that the USFS recently updated its methodology for estimating aboveground biomass (Westfall et al., 2024). However, we used the earlier methods to ensure backwards compatibility with our earlier work (Horn et al., 2018). Also, given that the new methods had a relatively small effect, e.g., increased aboveground carbon by 11.6% mostly from the addition of treetops and limbs (Westfall et al., 2024), we do not think these changes would materially affect our results. That said, future work will use the updated USFS estimates. We excluded trees that were recorded as dead at both measurement inventories and trees that were harvested from the survival analysis. We retained data for tree species that had at least 500 individual trees for both growth and survival analyses (as compared to Horn et al., 2018 which retained trees with at least 2,000 individuals), resulting in 145 species for survival and 141 species for the growth analysis prior to model selection.

#### 2.1.1 Spatial disaggregation

Regional variability of tree responses to N and S deposition can be evaluated by spatially grouping tree populations into ecoregions (Omernik and Griffith, 2014; Bailey, 2016). Ecoregions are ecosystems of regional extent, and spatial boundaries are identified by abiotic and/or biotic characteristics. Available ecoregion categorization schemes in the U.S. include the Environmental Protection Agency’s Omernik classifications (Omernik and Griffith, 2014; Omernik, 1987) and Bailey’s ecoregions developed for the USFS (Bailey, 2016). We used Bailey’s ecoregions, similar to earlier work (Potter and Woodall, 2014), to delineate regions in our analysis. Regions (referred to as “Division”) are subdivided based on precipitation and temperature levels. There are 11 Divisions in the conterminous U.S. (Supplementary Figure S1).

### 2.2 Modeling tree species growth and survival responses within Divisions

#### 2.2.1 Responses

As was done previously in Horn et al. (2018), we calculated tree growth rates (kg C tree^−1^ yr^−1^) as the difference in aboveground C between the latest and first live measurement of every tree and divided by the elapsed time between measurements to the day to limit pseudo-replication. Similarly, probability of tree survival [P(s) 10-yr^−1^] was calculated from the first live measurement to the last live measurement or to the first measurement recorded as dead for each tree inventoried. This results in sampled trees with varying periods between measurements ranging from about 5–10 years (8.2 years ± 2.9 years; mean ± S.D.).

#### 2.2.2 Predictors

We included up to six factors influencing growth or survival: mean annual temperature (K) (*T*), total annual precipitation (dm) (*P*), mean annual total nitrogen deposition (kg N ha^−1^ year^−1^) (*N*), mean annual total S deposition (kg S ha^−1^ yr^−1^) (*S*), tree size (*m*), and plot-level competition (*C*). To obtain total N and S deposition rates for each tree, we used spatially modeled N and S deposition data from the National Atmospheric Deposition Program’s Total Deposition Science Committee (Schwede and Lear, 2014). We averaged annual N and S deposition rates from the 1^st^ year of measurement to the last year of measurement for every tree so that each tree had an individualized average N deposition based on the measurement years, and each species had an individualized range of N deposition exposure based on its geographic distribution. For example, censuses for red maples may have occurred in 2005 and 2010 in one state and in 2006 and 2011 in another state, therefore, average N deposition was averaged over a different set of years for the same species depending on plot location.It should be noted that atmospheric N and S deposition was likely declining over the period of record (Benish et al., 2022), but the trend in deposition was not used in the modeling, only the temporal average over the first and last measurement period. We obtained annual mean temperature and precipitation values in a gridded (4 km × 4 km) format from the PRISM Climate Group at Oregon State (https://prism.oregonstate.edu/normals/) (Daly et al., 2008) for the contiguous U.S. and averaged between measurement periods for each tree in a similar manner. We assigned tree size (*m*) using aboveground tree C (previously described). Because these interpolated climate and deposition predictors were tailored to each plot in which a tree occurred, the years assessed varied by plot, but fell within the 2000–2014 range. Finally, we modeled competition between trees as a function of plot basal area (*BA*) and the basal area of trees larger than that of the tree of interest (*BAL*) similar to the methods of Pukkala et al. (2009) and Horn et al. (2018).

#### 2.2.3 Models and model selection

We developed multiple models to predict tree growth and survival. Our growth model assumes that there was a potential growth rate (*a*) modified by up to six predictors in our study: temperature, precipitation, N deposition, S deposition, tree size, and competition. The full growth model included all six terms (Equation 1 for the general form).


(1) $$G=a\times \text{competition}\times \text{temperature}\times \text{precipitation}\times S_{dep}\times N_{dep}$$

We modeled the size effect as a power function (z) based on the aboveground biomass (m^z^), while the climate factors (i.e., temperature, precipitation) were modeled as two-term lognormal functions. The two-term lognormal functions (e.g., t_1_ and t_2_, or p_1_ and p_2_, for temperature and precipitation, respectively) allow for flexibility in both the location of the peak (t_1_, p_1_, etc.) and the steepness of the curve on either side (t_2_, p_2_, etc.). Additionally, we modeled N deposition as a two-term lognormal function to allow for a portion of N deposition that had a positive effect and a portion with a negative effect, with a peak and steepness that could vary by species. Sulfur deposition in Horn et al. (2018) was modeled as a lognormal that was constrained to be negative. This was intentional in Horn et al. (2018) because allowing S deposition to have the same relationship with growth as N deposition aggravated any co-linearity these two covariates may have, and most evidence available points to a negative effect from S deposition through soil acidification. Furthermore, most terrestrial ecosystems are N or P limited, and S limitation is rare in natural ecosystems (Lebauer and Treseder, 2008; Elser et al., 2007). However, we discovered that constraining S to have a negative lognormal relationship could lead to unrealistically low critical loads (i.e., the minimum S deposition). Thus, we modeled S deposition two ways in this effort: (1) as a sigmoid function so that there could be a portion of S deposition with no effect followed by a decreasing portion that leveled off, and (2) as a lognormal function constrained to be negative as was done in Horn et al. (2018) for backwards compatibility. For the sigmoid representation, the portion where 50% of the decrease has occurred was governed by s_1_, the steepness of the decrease was governed by s_2_, and the plateau where the response levels off at high deposition was governed by s_3_. The full expression for the growth model with all potential terms is shown in Equations 2, 3 for sigmoid and lognormal S relationships, respectively.

We examined a total of seven different growth models of increasing complexity: (1) the intercept only model, (2) a model with only competition and climate terms (termed the “base model”), (3) the base model with a lognormal N function added, (4) the base model with a sigmoid S function added (Equation 2), (5) the base with a lognormal S function added, (6) the base model with lognormal N and sigmoid S functions added (Equation 2), and (7) the base model with lognormal N and lognormal S functions added (Equation 3).


(2) $$G=a\times m^{z}\times e^{\left(a_{2}BAL+a_{3}\ln(BA)\right)}\times e^{-0.5{\left(\frac{\ln\left(\frac{T}{t_{1}}\right)}{t_{2}}\right)}^{2}}\times e^{-0.5{\left(\frac{\ln\left(\frac{P}{p_{1}}\right)}{p_{2}}\right)}^{2}}\times e^{-0.5{\left(\frac{\ln\left(\frac{N}{n_{1}}\right)}{n_{2}}\right)}^{2}}\times \left[s_{3}+\left(\frac{1-s_{3}}{\left(1+{\left(\frac{S}{s_{1}}\right)}^{s_{2}}\right)}\right)\right]$$

(3) $$G=a\times m^{z}\times e^{\left(a_{2}BAL+a_{3}\ln(BA)\right)}\times e^{-0.5{\left(\frac{\ln\left(\frac{T}{T_{1}}\right)}{t_{2}}\right)}^{2}}\times e^{-0.5{\left(\frac{\ln\left(\frac{P}{p_{1}}\right)}{p_{2}}\right)}^{2}}\times e^{-0.5{\left(\frac{\ln\left(\frac{N}{n_{1}}\right)}{n_{2}}\right)}^{2}}\times e^{-0.5{\left(\frac{\ln\left(\frac{S}{s_{1}}\right)}{s_{2}}\right)}^{2}}$$

We estimated the annual probability of survival [P(s) 10-year^−1^] similarly to growth, except that the probability was a function of time and we explored two different representations for competition. The general form of the model is shown in Equation 4, and the full survival model in Equations 5, 6 for the two competition forms (only sigmoidal S form shown for simplicity).


(4) $$P(s)={\left[a\times \text{size}\times \text{competition}\times \text{temperature}\times \text{precipitation}\times N_{\text{dep}}\times S_{\text{dep}}\right]}^{\text{time}}$$

(5) $$P(s)={\left[a\times \left(1-zc_{1}e^{-zc_{2}\text{size}}\right)\times e^{-zc_{3}}\text{siz}e^{zc_{4}}\times e^{-br_{1}\left(BA_{\text{ratio}}^{br_{2}}\right)\left(BA^{br_{3}}\right)}\times e^{\frac{-1}{2}{\left(\frac{\ln\left(\frac{T}{t_{1}}\right)}{t_{2}}\right)}^{2}}\times e^{\frac{-1}{2}{\left(\frac{\ln\left(\frac{P}{p1}\right)}{p_{2}}\right)}^{2}}\times e^{\frac{-1}{2}{\left(\frac{\ln\left(\frac{N}{n_{1}}\right)}{n}\right)}^{2}}\times \left(S_{3}+\frac{1-s_{3}}{1+{\frac{S}{S_{1}}}^{S_{2}}}\right)\right]}^{\text{time}}$$

(6) $${\left.P(s)=\left[a\times e^{\frac{-1}{2}\left(\frac{\ln\left(\frac{\text{size}}{z_{1}}\right)}{z_{2}}\right)}\times e^{\frac{-1}{2}{\left(\frac{\ln\left(\frac{BA}{ba_{1}}\right)}{ba_{2}}\right)}^{2}+\frac{-1}{2}{\left(\frac{\ln\left(\frac{BAL+1}{bl_{1}+1}\right)}{b_{2}}\right)}^{2}}\times e^{\frac{-1}{2}{\left(\frac{\ln\left(\frac{T}{t_{1}}\right)}{t_{2}}\right)}^{2}}\times e^{\frac{-1}{2}{\left(\frac{\ln\left(\frac{P}{p_{1}}\right)}{P_{2}}\right)}^{2}}\times e^{\frac{-1}{2}{\left(\frac{\ln\left(\frac{N}{n_{1}}\right)}{n}\right)}^{2}}\times \left(S_{3}+\frac{1-s_{3}}{1+{\frac{S}{S_{1}}}^{S_{2}}}\right)\right]\right]}^{\text{time}}$$

We examined a total of 13 survival models, the same combinations of models for growth but with two different competition representations (note the intercept-only model only has one representation since competition is not included).

Parameters for each of the 7 growth and 13 survival models above were fit for a given species using maximum likelihood estimates through simulated annealing with 100,000 iterations via the “likelihood” package (Murphy, 2022) in R version 4.1.3 (R Core Team, 2022). We calculated Akaike’s Information Criteria (AIC) for all models and selected the model with the lowest AIC for use in our analysis according to the following conditions. If there were multiple candidate models with ΔAIC ≤ 4.0, we selected the model with the lowest AIC that included separate parameters for N and S deposition. We excluded models from species × Divisions if the candidate model set included only models with combined N and S parameters and N and S and the correlation between N and S deposition was high (r ≥ 0.7). This resulted in the exclusion of *n* = 32 species × Divisions for growth and *n* = 15 species × Division for survival (Supplementary Tables S3, S4). We report all selected models and estimated parameters, including parameters for calculating N and S critical loads, in the online data repository (https://doi.org/10.23719/1529764).

#### 2.2.4 Estimating critical loads

From the selected overall models, we may estimate critical loads for N and S. For N, the critical load is best estimated as the n_1_ term, which is the point above which growth or survival begins to decline. For S, the estimate of the critical load depended on whether the sigmoid or lognormal function is used. For lognormal S-responses, the critical load is s_1_ (as with n_1_ for N). For sigmoid S-responses, the critical load is not directly estimated in the simulated annealing process. The s_1T_ term is the 50% reduction mark, which is too high for the critical load (the s_2T_ term governs the steepness of the curve and the s_3T_ term governs the bottom asymptote). Thus, for sigmoid S-responses the critical load is estimated *post-hoc*. The sulfur term (Equation 7) is a multiplier from 0–1 that represents the fractional reduction (*f)* in growth (or survival) given a rate of S deposition.

Thus, we assume a level of *f*, set Equation (7) equal to 1-*f*, and solve for S to estimate the CL:

(7) $$\frac{1-s_{3}}{\left(1+{\left(\frac{S}{s_{1}}\right)}^{s_{2}}\right)}=1-f$$

(8) $$S(\text{critical}\;\text{load})=s_{1T}*\sqrt[{s_{2T}}]{\frac{f}{1-f-s_{3T}}}$$

Common choices are a very small positive number (e.g., 0.001, for the critical load) or 0.01 for survival and 0.05 for growth (for a 1% or 5% reduction in survival or growth, respectively). We report n_1_, s_1_, and s_1T_ in the Supplementary Tables S6–S9 and all parameters from the selected models are included in the datafile (see Section Data availability statement).

### 2.3 Analyzing variation in tree growth and survival responses

#### 2.3.1 Responses

First, we used Fisher’s exact test to examine whether there were differences in the distribution of patterns of growth and survival responses to atmospheric N and S deposition among Divisions. Then, given the variation in tree response across Divisions, we analyzed the biotic and abiotic conditions that lead to vulnerable responses for species across Divisions. Because the derivation of the curves above were performed by Division, the Division was our analytical unit. Each species was assigned a single curve for each Division based on the best model (Section 2.3), leading to 241 individual responses for growth (i.e., species × Division combinations) and 268 individual responses for survival (Supplementary Tables S3, S4). The sample sizes of trees differ for growth and survival responses, because to calculate a growth curve, the tree must be present in both censuses; however, a tree that died between two censuses would be included in a survival calculation. Thus, the number of species that met the *N* = 500 cutoff is lower for growth compared with survival. We used the shape of the response curve (i.e., monotonic increase, monotonic decrease, unimodal/sigmoidal, and flat) to infer an index for vulnerability, creating an ordered categorical response variable based on the shape (i.e., vulnerability: increase < flat < unimodal/sigmoid < decrease). The index of vulnerability was based on whether the species has some (unimodal/sigmoid) or all (decreasing) of the trees in that Division responding negatively to N or S deposition. Species that benefitted (increasing) were the least vulnerable, and species that were non-responsive (flat) were intermediate.

#### 2.3.2 Predictors

There are many potential climate and ecological factors associated with variation in response to N and S deposition that have been suggested in the literature or reported in earlier research (Thomas et al., 2010; Horn et al., 2018; Schulte-Uebbing et al., 2022). We focus on the same four environmental factors above (i.e., N deposition, S deposition, temperature, precipitation) plus three additional factors: soil pH, mycorrhizal association (AM or EcM), and deciduousness (evergreen or deciduous) (Figure 1). We used 30-year mean annual temperature (°C), 30-year mean annual cumulative precipitation (mm), soil pH, atmospheric N deposition from 2000–2012 (kg N ha^−1^ yr^−1^), atmospheric S deposition from 2000–2012 (kg S ha^−1^ yr^−1^). Since each species only has one response-shape per Division, predictors were characterized at the Division level by averaging across all of the FIA plots for which a species occurred. For categorical variables (mycorrhizal association and deciduousness), we assigned each species to a specific category, independent of Division.

##### Abiotic factors:

We extracted the 30-year mean annual temperature (MAT) and precipitation (MAP) (1991–2020) at the USDS FIA plot level using the PRISM climate normals (https://prism.oregonstate.edu/normals/) and deposition data from the National Atmospheric Deposition Program from 2000–2012 (Daly et al., 2008; Schwede and Lear, 2014). Likewise, we extracted soil pH estimates at the FIA plot level from the Gridded National Soil Survey Geographic Database (https://www.nrcs.usda.gov/resources/data-and-reports/gridded-national-soil-survey-geographic-database-gnatsgo). We determined mean climatic, soil pH, and deposition conditions for a species, within a forest Division and across the U.S., by averaging all interpolated plot-level temperature, precipitation, soil pH, and N and S deposition observations where a specific species was observed. We tested whether this was variation in these factors among Divisions using one-way Analysis of Variance (ANOVA) and Tukey’s Honest Significant Difference *post-hoc* tests.

##### Biotic factors:

For mycorrhizal association, we obtained data from Jo et al. (2019), which collated data from peer-reviewed journal publications. Only 3 of 141 species were not characterized as having associations with AM or EcM in our dataset (*Ailanthus altissima*–non-mycorrhizal, *Gledistia triacanthos*–EcM + AM, *Oxydendrum arboreum*–ericoid mycorrhiza). In our study, we updated the known association of *Ailanthus altissima* to AM based on recent literature (Huebner et al., 2007). Given *Glaedistia triacanthos* was first assigned AM, we re-ran models after assigning the species to EcM (Bainard et al., 2011), which did not result in significant changes to the conclusions. Finally, we removed *Oxydendrum arboreum* from our models because it was the only species with ericoid mycorrhizal fungal association. Next, we gathered data on deciduousness of each species using data from Horn et al. (2018) and supplementing the data with information from TRY species database (TraitID vegetative phenology) (https://www.try-db.org/TryWeb/Home.php) (Kattge et al., 2011).

Prior to analysis, we used Pearson correlation coefficients to assess multicollinearity between our predictor variables at the Division scale. Only two bivariate relationships were correlated above a threshold of |0.7| (Dormann et al., 2013) (mean N deposition and S deposition: *r* = 0.801, t = 22.39, df = 264, *P* < 0.01; soil pH and precipitation: *r* = −0.72, t = −17.03, df = 264, *P* < 0.01) (Supplementary Table S1). We decided to include these variables in our analysis as we used a hypothesis-driven model framework.

Our analysis ultimately included 138 species for survival and 123 species for growth models at the CONUS level due to missing covariate data or high correlations between N and S deposition. In two of the 11 Divisions, just one species met our criteria for inclusion and therefore we discuss our results both with and without inclusion of these Divisions (Division 32 Tropical/Subtropical Desert–*Juniperus osteosperma*; Division 41 Savannah–*Taxodium ascendens*).

This analysis uses some of the same variables in two distinct ways to address two different research questions. In Section 2.2, we use mean N deposition, S deposition, temperature, and precipitation between census intervals to derive the growth and survival responses (8.2 years ± 2.9 years; mean ± S.D.). In Section 2.3, we are interested in the broader patterns of how these responses are distributed across the landscape (e.g., are there more decreasing responses in areas with historically high N deposition?) and among a few key traits (e.g., are there more decreasing responses among deciduous spp.?). Since Divisions cross state-boundaries, which largely define the remeasurement periods in the FIA, we use longer-term mean values (30 and 12 years for climate and deposition data, respectively) instead of the mean values between censuses intervals for these same variables within Divisions.

#### 2.3.3 Models and model selection

To examine potential covariates associated with the observed variation in tree survival and growth responses to N and S deposition, we ran proportional odds ordinal logistic regressions. This form of regression is used to model the relationship between two or more categorical response variables and a combination of quantitative and categorical predictors. To run the ordinal regression, we used the function *polr*() in the “MASS” package (Ripley et al., 2013). We ordered the responses to N and S deposition from the least vulnerable to the most vulnerable (increase < flat < unimodal/sigmoidal < decrease). Ordinal logistic regressions use maximum likelihood estimation to determine the odds of outcomes relative to others as a function of predictor variables. In our case, we tested whether the odds of ordinal responses to increasing N and S deposition (increasing, flat or no response, unimodal/sigmoidal, decreasing) depend on climate normals (temperature, precipitation), edaphic (soil pH), average N and S deposition from 2000–2012, tree functional traits (mycorrhizal association and deciduousness) (Supplementary Table S1). In addition to these main effects, we included six hypothesis-driven first-order interactions: (1) temperature × precipitation, (2) temperature × N deposition, (3) soil pH × S deposition, (4) N deposition × S deposition, (5) N deposition × mycorrhizal association, and (6) soil pH × mycorrhizal association. Although we focus on these hypothesis-driven interactions, we included results from models with only main effects, models with only N or S deposition, and models with all possible interactions (“global” models) in Supplementary Tables S10–S13. We ran a total of four separate ordinal models: (1) survival response to N deposition, (2) growth response to N deposition, (3) survival response to S deposition, and (4) growth response to S deposition. We performed stepwise selection using the function stepAIC() in the “MASS” package (Ripley et al., 2013) on the regression models starting with all possible combinations of main effects and hypothesized first-order interaction terms, to determine our selected model based on AIC. Although we focus on results from models selected by the stepwise procedure, models with all hypothesized factors are included in the Supplementary Tables S10–S13. We additionally ran multinomial logistic regressions using the R packages “nnet” (Ripley et al., 2016), which enable the intercepts and slopes to differ for each unordered response to deposition and found similar results. We visualized model outputs and predicted probabilities using the R packages “sjPlot” (Lüdecke et al., 2021) and “ggplot2” (Wickham et al., 2019).

#### 2.3.4 *Post-hoc* phylogenetic analysis of models

We tested for non-independence in our model residuals from our stepwise selected models due to phylogenetic relatedness. We used the residuals from each ordinal regression and the phylogenetic supertree tree of the 311 species in the FIA database (Potter and Woodall, 2012) to estimate the phylogenetic signal, or Blomberg’s K* (Blomberg et al., 2003), using the R package “phytools” (Revell, 2012) following methods in Pacifici et al. (2017).

## 3 Results

### 3.1 Climate, soil, deposition, and tree functional trait differences among Divisions

**FIGURE 2:**
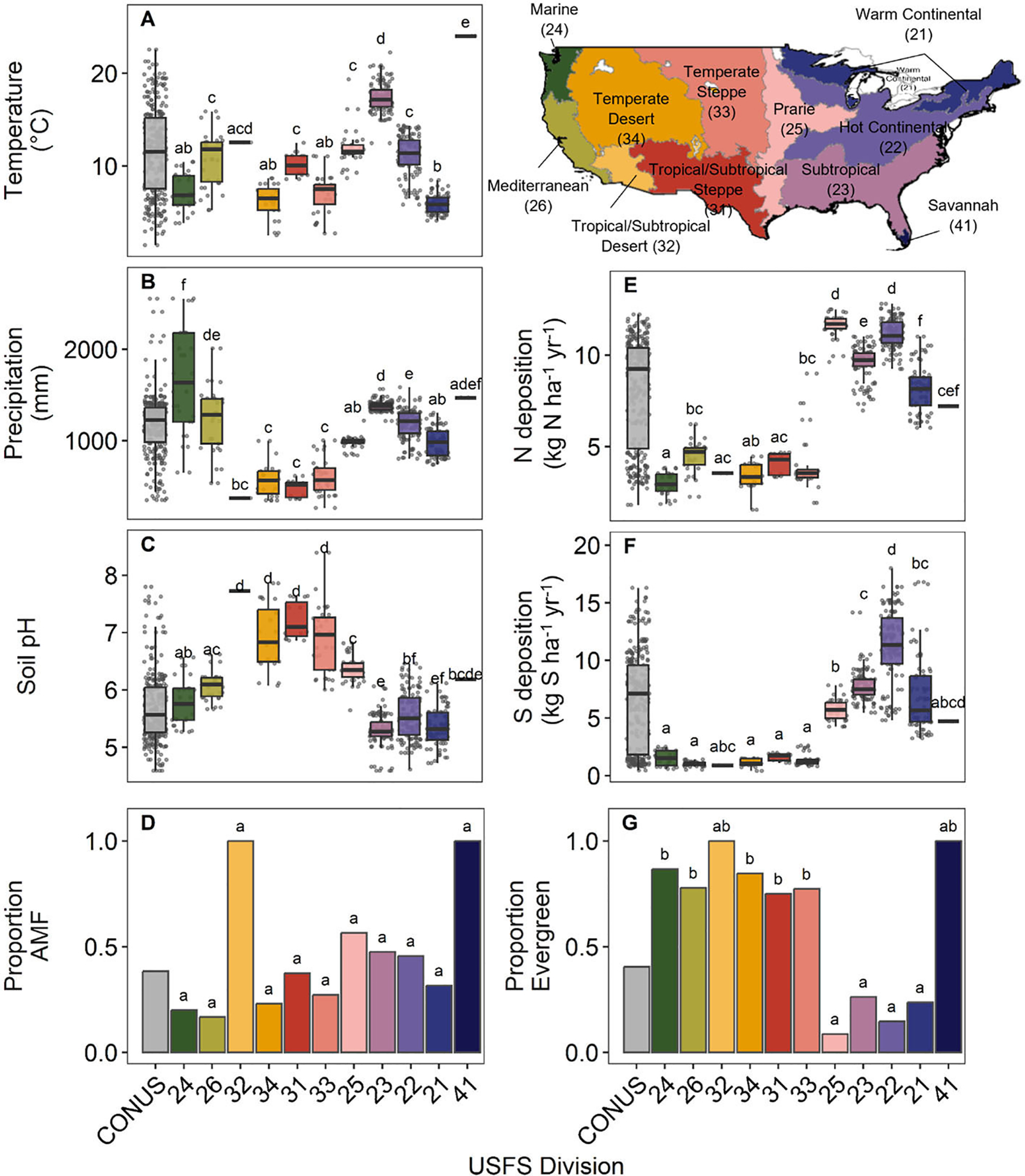
Climate, edaphic, and functional traits of trees in United States Forest Service (USFS) Divisions (ordered roughly west to east). **(A)** Thirty-year mean annual temperature (°C), **(B)** 30-year mean annual precipitation (mm), **(C)** mean soil pH, **(D)** proportion of tree species used in this analysis associated with arbuscular mycorrhizal fungi, **(E)** contemporary nitrogen deposition (kg N ha^−1^ yr^−1^), **(F)** contemporary sulfur deposition (kg S ha^−1^ yr^−1^), and **(G)** proportion of species in this study that are evergreen compared to deciduous. Lowercase letters indicate significant diferences between Divisions (Tukey’s honest significance test and Fisher’s exact test, *P* ≤ 0.05). Map lines delineate study areas and do not necessarily depict accepted national boundaries.

Mean annual temperature (°C), mean annual precipitation (mm), soil pH, N deposition (kg ha^−1^ y^−1^), and S deposition (kg ha^−1^ y^−1^) differed significantly among Divisions (Figure 2, Supplementary Table S2). Mean annual temperature among regions (ANOVA: F_10,255_ = 115.4, *P* < 0.001) ranged from 5.9°C in Division 21 (Warm Continental) (*n* = 38) to 17.42°C in Division 23 (Subtropical) (*n* = 61) and 24.0°C in Division 41 (Savannah) (*n* = 1) (Figure 2A). Mean annual precipitation across Divisions was 991.1 ± 421.9 mm (mean ± S.D.) and was significantly lower in Divisions in the central U. S. (e.g., Divisions 31–34; ANOVA: F_10,255_ = 52.18, *P* < 0.001) (Figure 2B). Conversely, soil pH was highest in the central Divisions (Divisions 31–34), ranging from 5.3 ± 0.3 (mean ± S.D.) in Division 23 (Subtropical) to 7.7 in Division 32 (Tropical/Subtropical Desert) (only one species in this Division) and 7.72 ± 0.32 in Division 31 (Tropical/Subtropical Steppe) (ANOVA: F_10,255_ = 65.09, *P* < 0.001) (Figure 2C). Nitrogen and S deposition (kg h^−1^ y^−1^) showed similar patterns across Divisions, with lower deposition in the western U.S. and higher deposition toward the eastern U.S. (ANOVA N deposition: F_10,255_ = 250.4, *P* < 0.001; S deposition: F_10,255_ = 75.61, *P* < 0.001) (Figures 2E, F).

The number of species known to be associated with AM vs. EcM did not significantly vary among Divisions (Fisher’s exact test: adjusted P = 0.53). Across the U.S., our analysis included 56 species associated with AM and 90 species associated with EcM (38.35% AM vs. 61.64% EcM) (Figure 2D). The number of deciduous species compared to evergreen varied among Division, as fewer evergreen species were represented in eastern Divisions (e.g., Divisions 21, 22, 23, and 25; Fisher’s exact test: adjusted *P* ≤ 0.001) (Figure 2G). Overall, our analysis includes 87 deciduous and 59 evergreen species. Note that for Divisions 32 (Tropical/Subtropical Desert) and 41 (Savannah) in only a single species is represented (Figures 2D, G).

### 3.2 Tree growth and survival responses across Divisions

**FIGURE 3:**
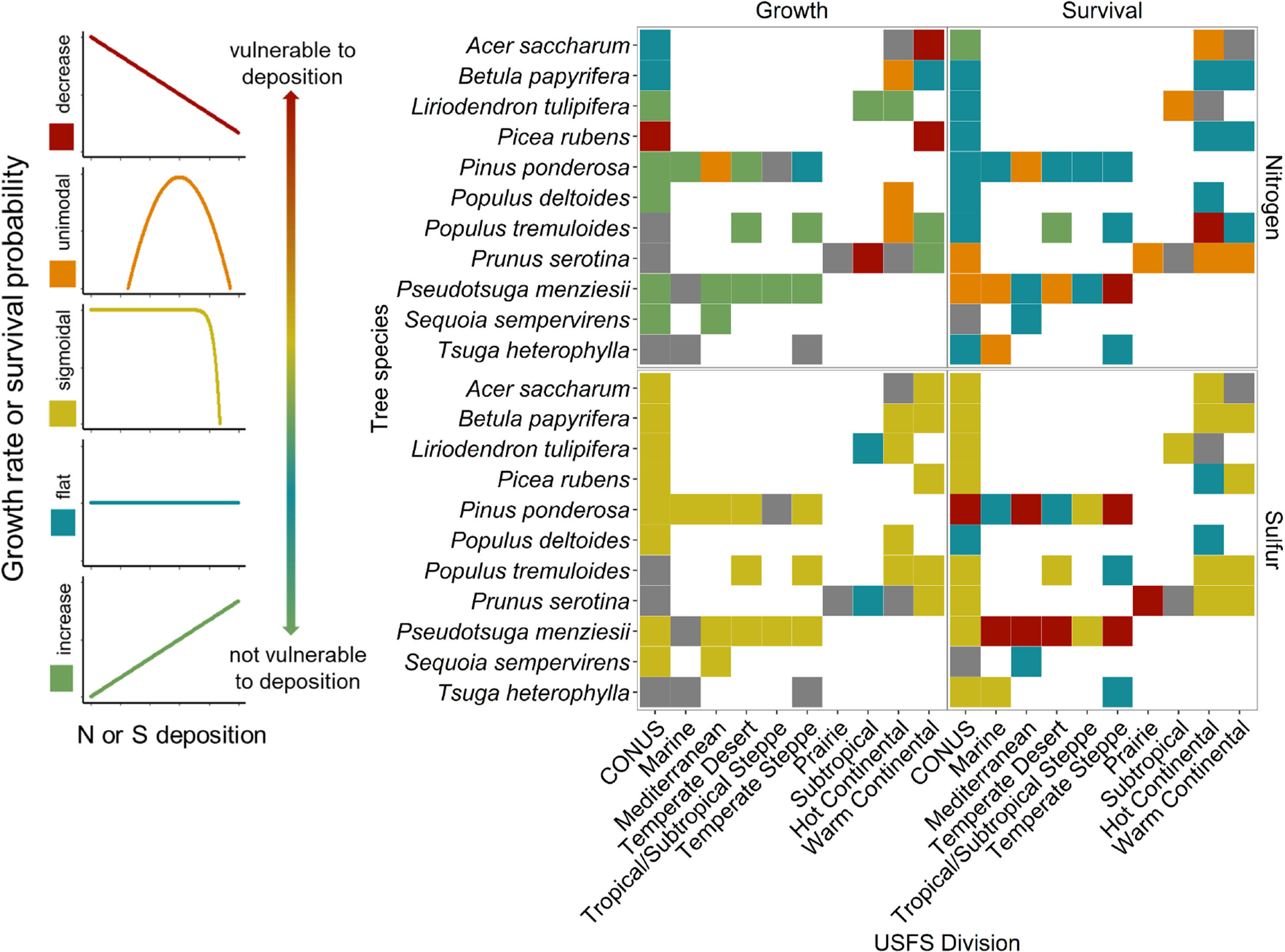
Species-specific growth and survival responses to nitrogen (N) or sulfur (S) deposition across the conterminous United States (CONUS) and United States Forest Service (USFS) divisions for a subset of species. Divisions are ordered west to east along the x-axis, and species are ordered alphabetically. The color of the square indicates the shape of the growth or survival responses to N and S (red = decrease, orange = unimodal, yellow = sigmoidal, blue = flat, green = increase, gray = omitted due to high correlation between N and S) and are ordered from most vulnerable (decrease) to least vulnerable (increase) to N or S deposition. All species-specific response curves are listed in Supplementary material 1.

**FIGURE 4:**
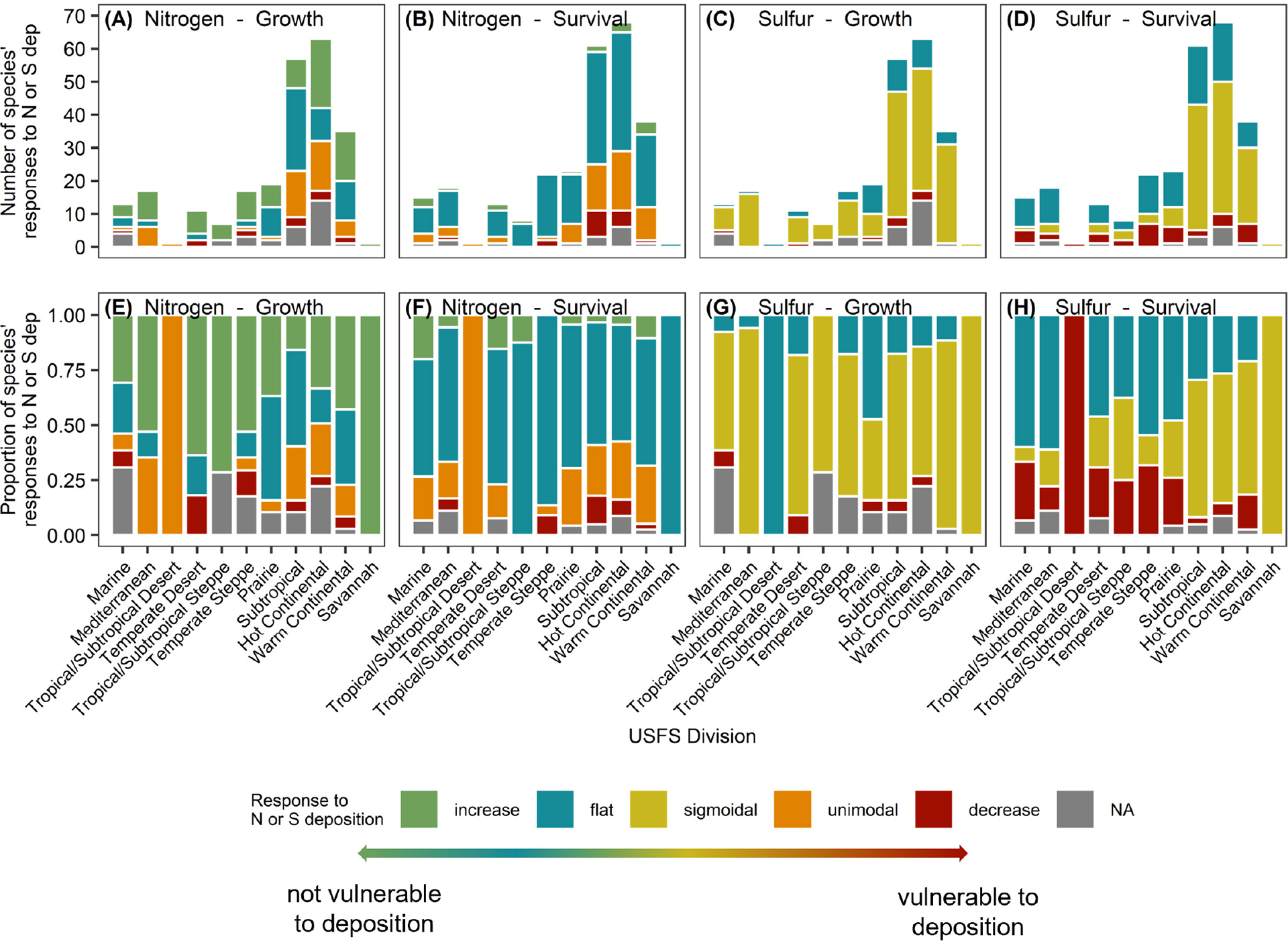
Distribution of responses across USFS Divisions (ordered roughly west to east). The number **(A–D)** and proportion **(E–H)** of tree species in each Division with flat (blue), increasing (green), decreasing (red), unimodal (orange, N deposition only), sigmoidal (yellow, S deposition only) aboveground tree growth (kg C tree^−1^ year^−1^), and survival [P(s) 10 yr^−1^] responses to increasing N deposition (kg N ha^−1^ yr^−1^) **(A, B, E, F)** and S deposition (kg S ha^−1^ yr^−1^) **(C, D, G, H)**. Note that we included a total of 141 species for growth relationships, and 145 species for survival relationships across the CONUS. Not applicable (gray) indicates that N deposition and S deposition were highly correlated and tree growth and survival responses to N or S alone were unable to be calculated.

Tree species responses to both N and S deposition varied by Division (Figures 3, 4). For a given Division, the number of tree species present varied, and therefore the number of curves that were dervied varied, as some forested regions are more diverse than others. Because of these differences in species numbers, analysis of *proportions* of curve shapes is more appropriate for analysis across Divisions. The proportion of curve shapes (increasing, flat or no response, unimodal/sigmoidal, and decreasing) were found to significantly vary among Divisions for species growth responses to N and survival responses to S deposition (Fisher’s exact test, *P* ≤ 0.05); however, variation of survival response to N and growth responses to S was not significantly different among Divisions (Figures 4A–H; Supplementary Tables S3–S5). Variation in the proportion of curve shapes suggests heterogeneity in the relative vulnerability of forests to atmospheric deposition. We also found that species tended to demonstrate more variable responses when distributed across more Divisions (Supplementary Figure S2). Less than 50% of species occurring in three or more Divisions had a consistent response curve shape for survival and growth response to N or S deposition (26.3 % for N-growth, 26.7% for N-survival, 47.4% for S-growth, and 16.7% for S-survival) (Supplementary Figure S2). We were unable to assign a growth or survival response to 18 and eight species, respectively, at the CONUS level due to high correlations between N and S deposition across CONUS (Figure 4, Supplementary Figure S3; Supplementary Tables S3–S5).

#### 3.2.1 Tree growth response to nitrogen deposition and covariates

Across the CONUS, many species showed an increase in growth rate in response to increasing N deposition (39.0% increase, 24.8% flat, 18.4% unimodal, and 5.0% decrease) (Supplementary Figure S3; Supplementary Tables S3–S5). There were significant differences in the proportion of the curve shapes in response to N deposition among Divisions (Fisher’s exact test, *P* = 0.003); and this pattern remained even after removing Divisions 32—Tropical/Subtropical Desert and 41—Savannah, which each only had one species with data meeting our sample size thresholds (Fisher’s exact test, *P* = 0.001). There were no obvious geographic patterns indicating a mechanism driving the shifts in the proportion of curve shapes. Out of the 58 species assessed that were in more than one Division, 21 had consistent responses and 37 had variable responses (Supplementary Figure S2A; Supplementary Table S5). Species present in more Divisions were more likely to show variable responses (Supplementary Figure S2A). Approximately 36.2% of species present in two or more Divisions had consistent growth responses and this fraction decreased as species were found in more Divisions (e.g., 26.3% of species showed consistent responses if present in three or more Divisions (Supplementary Figure S2A). For example, *Abies concolor* (white fir) was assessed for three different Divisions (24, 26, 34) and was found to increase growth with N deposition in all three, while *Quercus alba* (white oak) was characterized in three different Divisions and increased in one (Division 21), flat or no response in one (Division 23), and a unimodal relationship in the third one (Division 22) and increased when all individual trees were aggregated for a CONUS-level response curve (Supplementary Table S5). Thus, variation among regional populations of a species appeared to be influenced by regional conditions, therefore context dependent. Species’ CLs varied across Divisions (Supplementary Table S6). For example, the N-CL for growth in *Pinus ponderosa* (ponderosa pine) varied from 8.7 kg N ha^−1^ yr^−1^ in Division 26 to 58.8 kg N ha^−1^ yr^−1^ in Division 24 (Supplementary Table S6).

**FIGURE 5:**
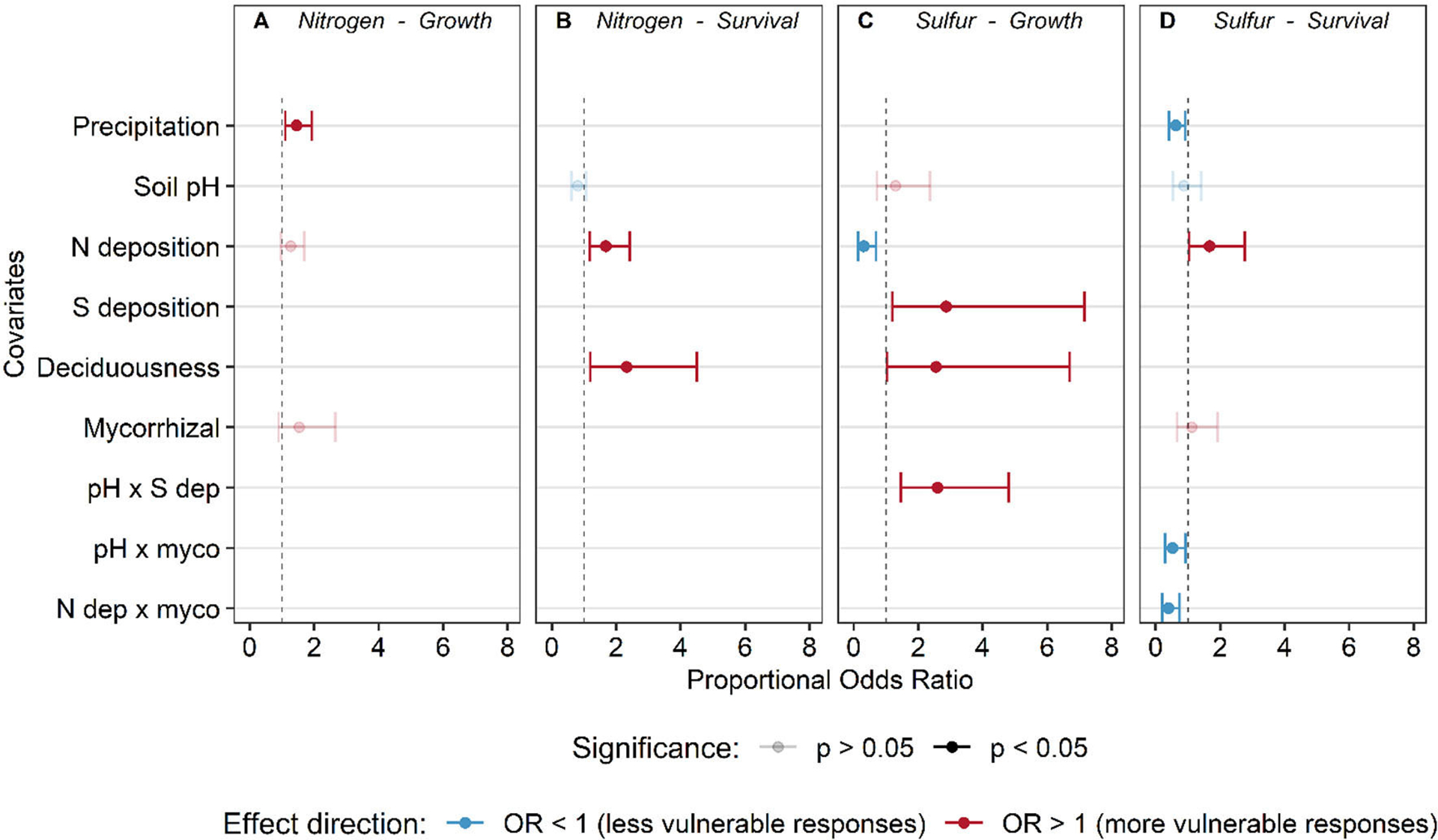
Proportional odds ratio ± 95% confidence intervals from the selected ordinal logistic regression models for tree growth **(A, C)** and survival **(B, D)** response to nitrogen **(A, B)** and sulfur deposition **(C, D)**. Odds ratios falling to the right of the dashed line indicate that with an increase in a covariate, the odds of a more vulnerable response (flat vs. increase, unimodal vs. flat, decrease vs. unimodal/sigmoidal) (red–odds ratio > 1) are increased, while points to the left indicate the odds of a less vulnerable shape are increased with a unit change in the covariate (blue–odds ratio < 1). Solid points indicate statistical significance (*P* ≤ 0.05), while transparent points are statistically non-significant (*P* > 0.05) but still in the best model via model selection and AIC.

**FIGURE 6:**
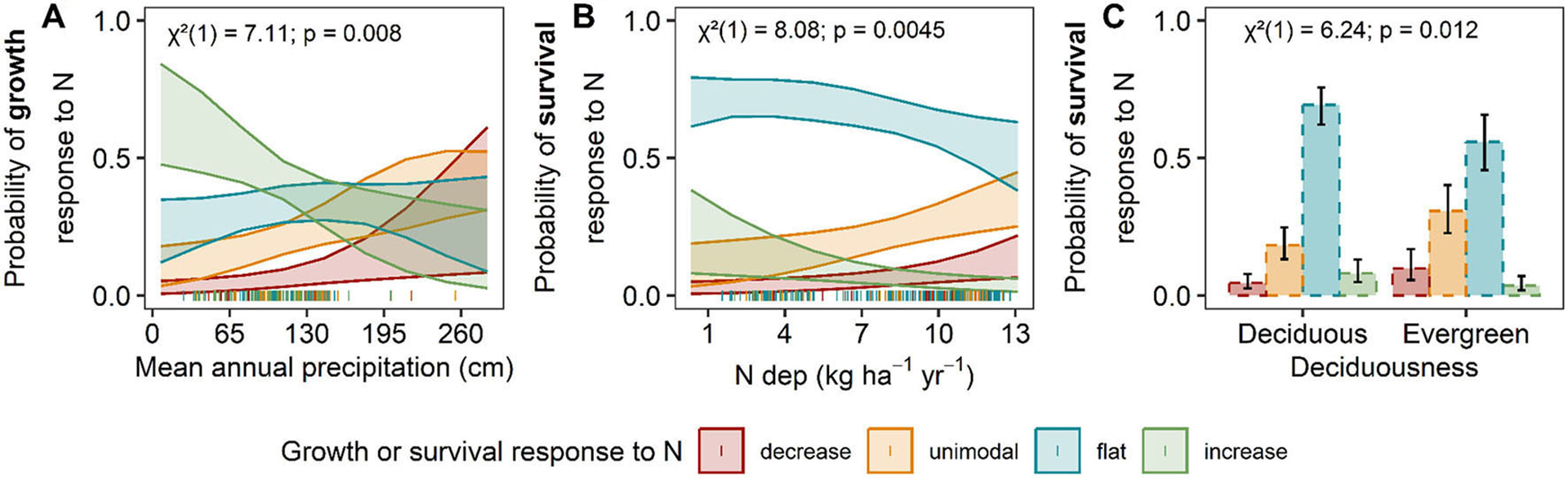
Predicted tree growth **(A)** and survival responses **(B, C)** to N deposition. Predicted relationship between the distribution of N-growth (solid lines) and N-survival (dashed lines) responses change along significant environmental factors. The only significant factor in the hypothesis-driven N-growth model was **(A)** mean annual precipitation (cm), and in N-survival models are **(B)** N deposition (kg N ha^−1^yr^−1^) and **(C)** deciduousness. The y-axis is the predicted probability of a decreasing (red), unimodal (orange), flat (blue), or increasing (green) N-growth or N-survival relationship as the environmental factor change. The shaded bands represent 95% confidence intervals. Chi-square (χ^2^), degrees of freedom, and *P*-values are reported for each covariate or interaction term in the upper left corner. Actual data points are plotted along the x-axis.

Ordinal logistic regressions revealed that MAP was the most influential factor affecting the predicted vulnerability of tree growth response to N deposition (Figure 5A, Supplementary Table S10; model AICc = 509.6, Nagelkerke’s R^2^ = 7.5%). Both the global and main effects models explained slightly more variation, but the ΔAIC between the selected model and the global and main effects models were large (ΔAIC > 7, Supplementary Table S10), suggesting an inflated R^2^ from the inclusion of additional terms in the non-selected models. The predicted frequency of vulnerable growth responses increases with increasing precipitation (i.e., higher probability of a decreasing response and lower probability of increasing response to N deposition) (Figure 6A).

#### 3.2.2 Tree survival response to nitrogen deposition and covariates

Although a positive response in growth rate was the most common response to increasing N deposition at the CONUS level, flat (54.8% of species) and unimodal (24.0% of species or 1.03 million measured trees) responses were the prevailing survival responses to N, consistent with results of Horn et al. (2018) (Supplementary Figure S3, Supplementary Table S3; 8.2% decreasing, 7.5% increasing, unable to assess response 5.5%). However, unlike growth responses, the distribution of survival responses to N deposition did not differ significantly among Divisions (Fisher’s exact test, *P* = 0.363; without Divisions 32 and 41, *P* = 0.339). Like growth responses to N (Section 3.2.1), there was variation in species’ CLs across Divisions (Supplementary Table S7). For example, the N-CL for survival in *Quercus velutina* (black oak) varied from 5.66 kg N ha^−1^ yr^−1^ in Division 23 (Subtropical) to 13.69 kg N ha^−1^ yr^−1^ in Division 25 (Prarie).

Species with larger ranges in our analysis (those present in three or more Divisions) were more likely to show variable survival responses to N deposition rather than a consistent response (73.3% of species with variable responses) (Supplementary Figure S2B). Out of the 70 species assessed that were in more than one Division, 29 species had consistent responses and 41 had variable responses (Supplementary Table S5; Supplementary Figure S2B). Significant covariates for the relationship between species’ survival responses to increasing N deposition include N deposition (χ^2^ = 8.08, *P* = 0.004) and deciduousness (χ^2^ = 6.42, *P* = 0.012) (Figures 5, 6B, C, and Supplementary Table S11; AICc: 494.3; Nagelkerke’s R^2^ = 7.3%). The global model, which included all factors and possible interactions, and the main effects models were not competitive with the selected hypothesis-driven model (ΔAIC > 4, Supplementary Table S11). From the selected model, we found that as N deposition increased, the proportion of vulnerable responses (decreasing and unimodal) increased (Figure 6B). In addition, evergreen species were 2.3 times (95% CI: 1.19–4.5 times) more likely to exhibit more vulnerable responses (decreasing or unimodal) relative to deciduous species (Figure 6C).

#### 3.2.3 Tree growth response to sulfur deposition and covariates

The most common growth response to increasing S deposition was a negative sigmoidal (69.5% of species), followed by flat (12.1% of species) and decreasing responses (5.7%) (Supplementary Figure S3; Supplementary Table S4). Horn et al. (2018) did not include a sigmoidal response function, and thus a negative response was more common [of 74 species meeting variance inflation factor (VIF) thresholds, 45% demonstrated decreasing growth responses and 55% showed no response]. For most species, the sigmoidal response provided a better fit for the relationship between sulfur and growth/survival than a linear decline (Figures 4C, D, G, H). Tree growth responses to S deposition did not significantly vary among Divisions (Fisher’s exact test, *P* = 0.092; without Divisions 32 and 41, *P* = 0.120), and tree species present in three or more Divisions were as likely to have a consistent response across their range as a variable response (55.2% consistent, 44.8% variable) (Supplementary Figure S2C). Out of the 58 species assessed that were in more than one Division, 32 had consistent responses and 26 had variable responses (Supplementary Figure S2C; Supplementary Table S5).

**FIGURE 7:**
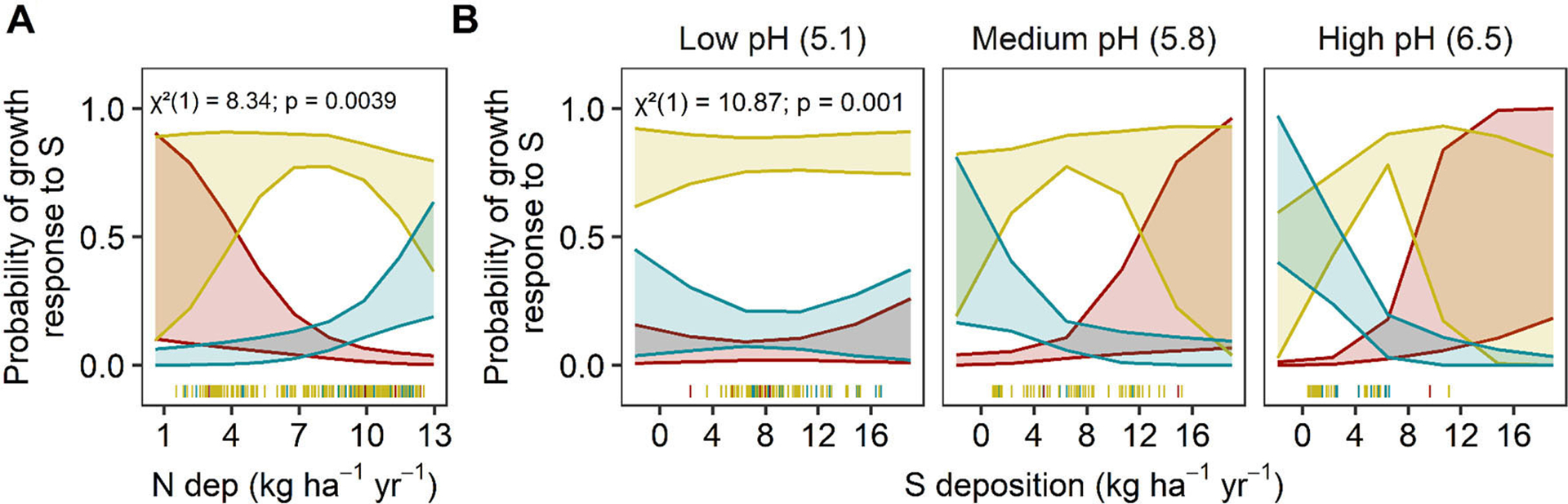
Predicted tree growth responses to S deposition. Predicted relationship between the distribution of tree species S-growth responses change along significant environmental factors. Significant main effects of **(A)** N deposition (kg N ha^−1^yr^−1^) and interactions between **(B)** S deposition (kg S ha^−1^yr^−1^) and soil pH are presented. The y-axis is the predicted probability of a decreasing (red), sigmoidal (yellow), or flat (blue) S-growth relationship as environmental factors change. The shaded bands represent 95% confidence intervals. Bins for soil pH are the mean values ± 1 S.D. Chi-square (χ^2^), degrees of freedom, and *p*-values are reported for each interaction term in the upper left corner of each plot. Actual data points are plotted along the x-axis.

The most important predictors for S-growth response were the N and S deposition main effects (N dep: χ^2^ = 8.34, *P* = 0.003; S dep: χ^2^ = 0.02, *P* = 0.031), deciduousness (χ^2^ = 4.09, *P* = 0.04), and the interaction between soil pH and S deposition (χ^2^ = 10.86, *P* = 0.0009 (Figure 7, Supplementary Figure S4, Supplementary Table S12; AICc: 266.18; Nagelkerke’s R^2^ = 13.8%). There were no other competing models (global or fixed effects models) that differed meaningfully within a ΔAIC of 2.0 (all ΔAIC > 12.0, Supplementary Table S12). With increasing N deposition, a less vulnerable response (flat vs. sigmoidal, sigmoidal vs. decreasing) was 31.4% (95% CI: 14.1–69.1%) more likely than a vulnerable response (decreasing vs. sigmoidal, sigmoidal vs. flat) (Figure 7A). The interaction between soil pH and S deposition suggested that at low pH (~5.1), sigmoid relationships dominate and are unaffected by S deposition, but at medium (~5.8) or high pH (~6.5) vulnerability increased with increasing S deposition. It is important to interpret these interactions with caution, as most plots with higher S deposition also had lower soil pH (Figure 7B).

#### 3.2.4 Tree survival response to sulfur deposition and covariates

Like growth responses to S deposition, just over half of tree species demonstrated a sigmoidal response in survival probability (52.1% of species) followed by a flat (34.2%) and decreasing responses (8.2%) across CONUS (Supplementary Figure S3; Supplementary Table S4). Of the 74 tree species meeting the VIF threshold in Horn et al. (2018), the majority demonstrated a decrease in survival probability to increasing S deposition (57% decreasing, 43% flat or no response). The distribution of tree survival responses to S deposition differed among Divisions (Fisher’s exact test, *P* < 0.001; without Divisions 32 and 41, *P* < 0.001). Out of the 70 species assessed that were in more than one Division, 21 had consistent responses and 49 had variable responses (Supplementary Figure S2D; Supplementary Table S5). Most species’ survival response to S deposition varied across their range, if present in three or more Divisions (83.3% variable response, 16.7% consistent response).

**FIGURE 8:**
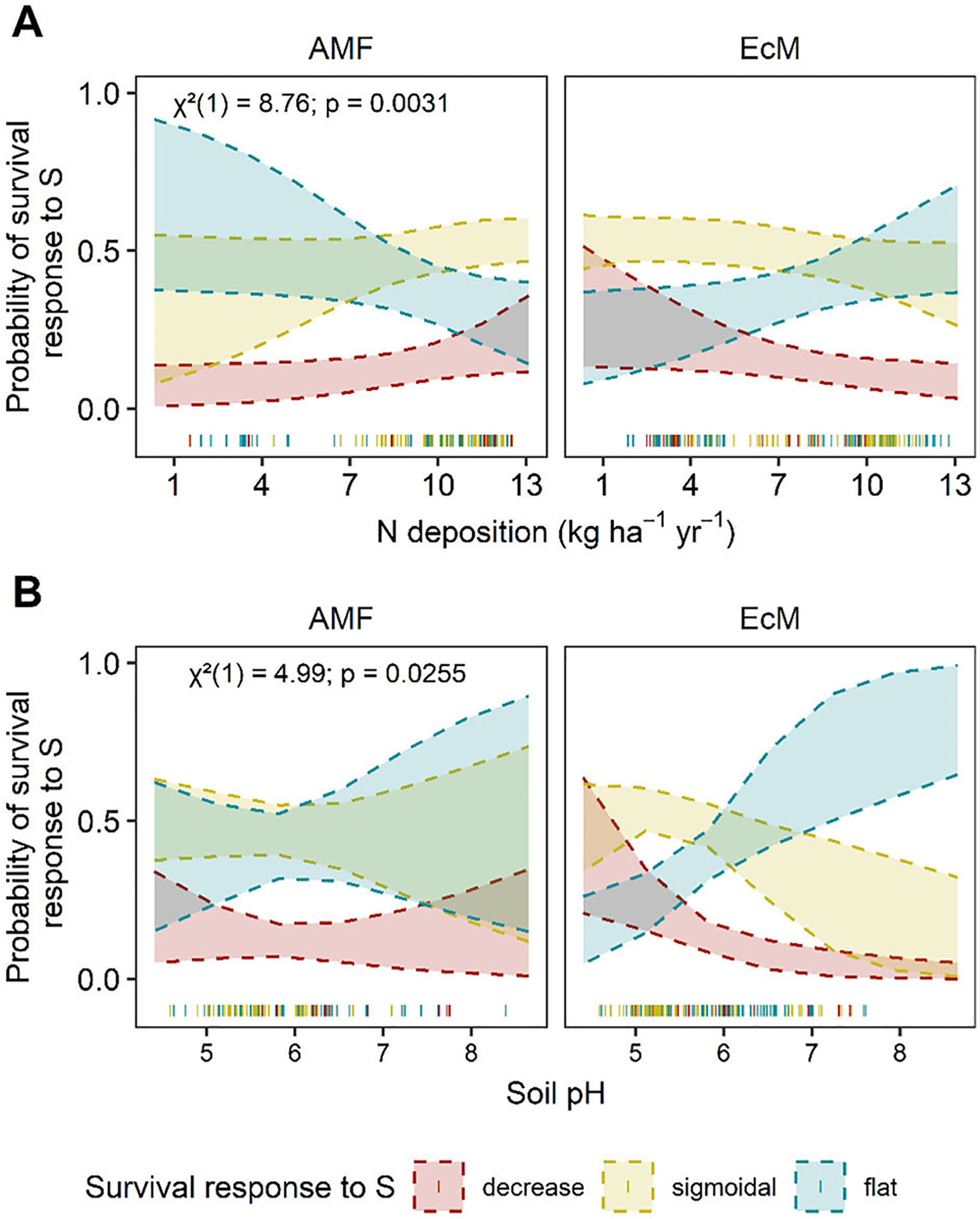
Predicted AM and EcM-associated tree survival responses to S deposition. Predicted relationship between the distribution of trees associated with arbuscular mycorrhizal fungi (AM) or ectomycorrhizal fungi (EcM) survival (P(s) 10 yr^−1^) responses change along significant environmental factors. Significant interactions between **(A)** N deposition (kg N ha^−1^ yr^−1^) x mycorrhizal association and **(B)** soil pH x mycorrhizal association are presented for S-survival. The y-axis is the predicted probability of a decreasing (red), sigmoidal (yellow), or flat (blue) S-survival relationship as the environmental factors changes. Shaded bands represent the 95% confidence intervals. Chi-square (χ^2^), degrees of freedom, and *P*-values are reported for each interaction term in the upper left corner. Actual data points are plotted along the x-axis.

There were three significant main covariates in the ordinal regression model predicting the frequency of survival responses to S deposition. Overall, we found that precipitation (χ^2^= 6.02, *P* = 0.014) and two interactions between known mycorrhizal association and either N deposition (χ^2^ = 8.76, *P* = 0.003) or soil pH (χ^2^ = 4.99, *P* = 0.025 were correlated with survival response to S deposition (Figures 5D, 8, and Supplementary Figure S5; Supplementary Table S13; AICc: 502.3; Nagelkerke’s R^2^ = 6.8%). There were no competing models (all ΔAIC > 6, Supplementary Table S13). The probability of a decreasing response declines with increasing N deposition for tree species associated with EcM, while remains relatively constant or slightly increases over N deposition gradients for species associated with AM (Figure 8A). In addition, the predicted probability of a less vulnerable response (no response) increases with soil pH relative to sigmoidal and decreasing responses for EcM-dominated trees (Figure 8B).

#### 3.2.5 *Post-hoc* phylogenetic analysis of models

Values of Blomberg’s K* range from 0 to infinity, with 0 suggesting no phylogenetic signal in the trait and 1 indicating high phylogenetic signal. For all selected models, K values ranged from 0.13 to 0.16 (*P* ≥ 0.05), suggesting non-significant phylogenetic patterns in the residuals of our selected models (Nitrogen–Growth: K = 0.161, *P* = 0.424; Nitrogen–Survival: K = 0.142, *P* = 0.434; Sulfur–Growth: K = 0.159, *P* = 0.330; Sulfur–Survival: K = 0.139, *P* = 0.485). We additionally ran these analyses using the residuals from intercept-only models, as mycorrhizal association and deciduousness likely have some phylogenetic signal and account for variation in our selected models. Using residuals from intercept-only models, we again found non-significant phylogenetic patterns (Nitrogen–Growth: K = 0.192, *P* = 0.087; Nitrogen–Survival: K = 0.143, *P* = 0.377; Sulfur–Growth: K = 0.147, *P* = 0.576; Sulfur–Survival: K = 0.130, *P* = 0.647).

## 4 Discussion

### 4.1 Characterizing variation in tree responses across Divisions

Here, we build on the work of Horn et al. (2018), which developed tree growth and survival responses to gradients of N and S deposition across the CONUS. In this study, we evaluated whether subdividing the species into sub-populations based on regional-scale spatial boundaries would reveal variation in the shape of the response curve (increasing, decreasing, unimodal/sigmoidal, and flat). Our analytical approach used Division-level ecoregions based on Bailey (2016)’s hierarchy in which spatial boundaries are defined by precipitation and temperature regimes. For the tree species in more than one Division, most had variable responses across their range. Overall, we found statistically significant differences in the proportion of tree species that have a given response curve shape [decreasing, unimodal (N)/sigmoidal (S), flat, and decreasing] among the Divisions for growth response to N deposition and survival response to S deposition, but not for survival response to N deposition and growth response to S deposition. Variation in response curves among Divisions could be a result of different species composition in the Divisions or variation in the shape of the response curve of individual tree species when in different Divisions. Approximately 60% of species that occur in two or more Divisions (range: 44.8%−70%) have a variable growth and survival responses to N and S deposition. However, when a species occurs in three Divisions, more than 70% of species (range: 52.6%−83.3%) have variable response shapes across those Divisions, indicating that more broadly distributed species tend to have regional variation in N and/or S response curve shape.

While tree response curves calculated for a given species at a national scale are important, the national aggregation obscures how species vary in response to N and S deposition across multiple ecoregions sometimes masking negative impacts of air pollution. For example, *Ulmus americana* (American elm) in Horn et al. (2018) and here demonstrated monotonically increasing growth rate responses with N deposition at the CONUS level. However, we found that in the southeast (Division 23–Subtropical), there was a critical load estimated at 28.5 kg N ha-^1^ yr^−1^. This was the only Division where such a critical load would have been detected, as the range of N deposition (4.8–55.4 kg N ha^−1^ yr^−1^) was higher than other Divisions with *U. americana* (3.6–23.9 kg N ha-^1^ yr^−1^ across the other three Divisions). Thus, a CONUS-wide approach may obscure regional variation in the response. It is possible that the variation across region for *U. americana* and other species may simply be explained by different regions experiencing different levels of deposition (e.g., a unimodal relationship at the CONUS level subdivided into an increasing and decreasing relationship among two regions). However, the presence of several non-deposition factors in the ordinal regression suggests the relationships are more complex.

#### 4.1.1 Growth and survival responses to nitrogen deposition

Nitrogen addition is known to increase tree growth, because N is often the most limiting nutrient in terrestrial ecosystems (Etzold et al., 2020). Tree growth response curves to N deposition at the CONUS level reported by Horn et al. (2018) indicate the largest proportion of tree species’ growth rates do not respond to increasing N deposition (45.1% of species), while fewer species show increasing (28.2%), unimodal (23.9%), and decreasing (2.8%) growth rate responses. In comparison, our CONUS-level analysis found that positive growth response relationships were the most common response for species (39.0% increase, 24.8% flat, 18.4% unimodal, 4.9% decrease, and 12.8% unable to determine due to N and S correlation). Our results may differ from those in Horn et al. (2018), as our analysis included twice as many species due to our lower threshold for species inclusion (500 trees in this analysis vs. 2,000 trees in Horn et al., 2018). Our results are more consistent with the widespread observation of N limitation or co-limitation in terrestrial ecosystems (Lebauer and Treseder, 2008; Elser et al., 2007). Thus, it appears that the higher proportion of non-responding species in Horn et al. (2018) could be the result of contrasting relationships among Divisions canceling out. For example, *Acer negundo* (boxelder) growth was unaffected by N deposition (flat relationship) when individuals are aggregated at the national scale here and in Horn et al. (2018), but across the three Divisions it had an increasing (Division 25–Prairie), flat (Division 23–Subtropical), and decreasing (Division 22–Hot Continental) relationship (Supplementary Table S5). This did not appear to be driven by the range of exposure to N deposition, which was similar across Divisions (Division 25: 6.9–21.9 kg N ha^−1^ yr^−1^; Division 23: 5.3–31.1 kg N ha^−1^ yr^−1^; and Division 22: 5.7–26.5 kg N ha^−1^ yr^−1^). Thus, it appears that many of the non-responsive species in Horn may be masking regional variation due to counteracting regional responses.

Like Horn et al. (2018), many of the tree species in our study showed a flat survival probability response to increasing N deposition at the CONUS level (55% in Horn et al., 2018 and 54.8% here). Furthermore, we did not find a significant difference in the distribution of survival response curves to N deposition (e.g., increasing, flat, unimodal, and decreasing) among Divisions, meaning that across Divisions there was no statistical difference in the proportion of species demonstrating decreasing response curves vs. any other response curve (ignoring the two Divisions that only had one species analyzed). This is supported by the observation reported earlier that there are species that are sensitive and tolerant to N deposition nearly everywhere in the CONUS (Coughlin et al., 2023; Horn et al., 2018). That said, species that demonstrated decreasing survival responses with N deposition occurred predominantly in the southern Divisions of the eastern U.S. (8.2% of species aggregated at the CONUS), with a few additional species in the West.

#### 4.1.2 Growth and survival responses to sulfur deposition

Here, we restricted S deposition tree growth and survival responses to only include decreasing, sigmoidal, and flat curves, as S is not known to positively affect growth and survival rate responses. Unlike Horn et al. (2018) which only included monotonically negative growth and survival rate responses to S deposition, most tree species, when aggregated across the CONUS, demonstrated a sigmoidal growth and/or survival response to increasing S deposition (69.5% of species and 52.1%, respectively), by which growth and survival was unaffected by very low S deposition until deposition increased above some threshold. This was much more ecologically realistic compared with the response curves in Horn et al. (2018), because most soils have at least some buffering capacity to acid deposition. Thus, the critical loads for such species would increase from the minimum S deposition (in Horn et al., 2018) to some threshold estimated from Equation 8. The updated response curves in this paper can provide more information to calculate critical loads for regional populations of trees that are useful for decision makers. Of the species that occurred in two or more Divisions, 55.2% had a consistent growth response. Most of the tree species which exhibited a strictly decreasing growth response to S deposition occurred in five Divisions including the eastern U.S (Divisions 22–Hot Continental and 23–Subtropical), the midwest (Division 33–Temperate Steppe and 25–Prarie), and the northwest (Division 24–Marine) (Supplementary Table S5).

#### 4.1.3 Spatial variation in tree responses and critical loads

We used Bailey’s Division-scale ecoregions (Bailey, 2016) because that is the system that the USFS uses to categorize forests regionally, and we wanted to maximize the management utility of this work to potential decision makers who manage U.S. forests. However, there are other options for defining ecoregional boundaries that could aggregate individuals into different populations and potentially provide additional ecoregional patterns of tree N or S response curve shapes (Omernik and Griffith, 2014). For example, Omernik’s ecoregions have been used to evaluate ecosystem acidification (Scheffe et al., 2014), which could be useful future research that could evaluate tree response to combined total acidifying deposition (N + S deposition vs. S deposition), known to drive ecosystem acidification.

Here, we found evidence of variation in tree response curves to N and S deposition across Divisions. In this study, we did not directly compare CL values among Divisions, which may offer additional insight into species’ sensitivity to N and S. In a recent study, Coughlin et al. (2024) examined spatial variation in N CLs of 10 tree species. Coughlin et al. (2024) found small differences in N CLs when comparing across broad climatic regions, these authors did find variation in N CLs at a local scale. The N CLs reported in our Supplementary material are slightly higher than those in Coughlin et al. (2024) for species and Divisions that we can directly compare; for example, the survival CL for *Prunus serotina* (black cherry) in our study was 10.8–15.0 kg N ha^−1^yr^−1^ and in Coughlin et al. (2024) was 10.0–10.5 kg N ha^−1^yr^−1^.

### 4.2 Climate, soil, atmospheric deposition, tree functional traits associated with regional variation in growth, and survival responses

By and large, species-specific growth and survival responses were found to be regionally variable. Potential variability in these responses was further evaluated by ordinal-logistic regression models to determine statistically important abiotic and biotic covariates. The aim of this section is to provide over-arching findings as well as briefly describe hypothesized mechanisms for selected covariates (Figure 1).

#### 4.2.1 Climate predictors

Since Divisions are delineated based on climate (temperature and precipitation), it was not surprising that the 30-year average MAP was an important covariate influencing growth response to N and survival response to S deposition. Specifically, tree survival responses to S deposition were strongly influenced by MAP as species were more likely to demonstrate vulnerable responses (decreasing and sigmoidal responses) if they occur within more arid climates. Longer-term sulfur exposure has been correlated with increasing stomatal closure in plants, consequently decreasing drought tolerance (Savva and Berninger, 2010; McAinsh et al., 2002). The opposite association was found with N deposition, where forested species were less vulnerable (more increasing responses, fewer decreasing responses) under more arid conditions. In drier ecosystems, decomposition and mineralization can be slower due to less movement of microbes and enzymes in the soil matrices (Petraglia et al., 2019), leading to stronger N limitation and thus more positive responses to N deposition. We did not find evidence of a moderating influence of temperature, which could be due to several mechanisms. One possibility is that drought stress may have greater effects on morphological and physiological traits compared to temperature in sites with higher water limitation (Grossiord et al., 2017), potentially causing greater sensitivity to precipitation in growth and survival responses to atmospheric deposition.

#### 4.2.2 Soil pH

Although Divisions are not designed to capture soil pH heterogeneity, soil pH did vary among Divisions and was a significant factor in growth and survival response to S deposition. For instance, we found that species’ growth responses to S deposition are more vulnerable (decreasing and sigmoidal responses) at higher soil pH when S deposition was greater. This agrees with the extensive literature that S deposition decreases soil pH, and soil pH was associated with soil base cations status (BC/Al, Ca/Al and % base saturation), for which there are well documented thresholds that cause adverse tree response (Cronan and Grigal, 1995; Warfvinge et al., 1993). This increased probability of negative growth responses at higher soil pH may reflect that species/trees occurring on higher pH soils have less physiological resilience to declines in soil pH caused by higher rates of S deposition, while trees growing on more acidic soils (lower pH) are more adapted to those conditions (Smith et al., 2012) and less sensitive to further declines in pH caused by atmospheric deposition of S.

#### 4.2.3 Atmospheric N and S deposition

Contemporary N and S deposition were important predictors in three of four growth and survival responses (N-survival, S-growth, and S-survival). Background N or S deposition affected species’ responses, as the rate of deposition was expected to affect the response, but the presence of interactions suggests that the effects are more complex.

For responses to S, we found more vulnerable growth rate responses to increasing S deposition at low levels of N deposition, while sigmoidal responses were most common at median and higher levels of N deposition; however, there was a significant amount of variation at these lower levels. Low levels of N deposition may have a fertilizing effect on growth, such that the effect of increasing S deposition might be offset by the beneficial growth effects of covarying N deposition. Our data show at high levels of N deposition (e.g., >8 kg N ha^−1^ yr^−1^), trees may be less sensitive to additional S deposition, as soils may already be acidified. The onset of nitrate leaching in forests, an indicator of soil acidification, is often observed to occur between 7.5–10 kg N ha^−1^ yr^−1^ (Dise et al., 2009; Aber et al., 2003; Khalili et al., 2010). A weaker effect from S at high N deposition could be also indicative of local adaption or changes in community composition, whereby the populations or species in high N deposition areas are already selected to be acid-tolerant, leading to more flat relationships with S. Previous work using stable nitrogen isotopes in tree rings suggest that acidic deposition depresses N availability relative to plant demand through time (Sabo et al., 2020). Thus, sites where atmospheric N deposition was low and S deposition was high suffered from increased N limitation due to depressed mineralization in more acidified soils. At high N deposition sites where fertilization effects are stronger and base cation reservoirs are high (Lawrence et al., 2020), perceived sensitivity to S deposition was lower due to growth enhancements by high N additions.

#### 4.2.4 Deciduousness

Classifying trees based upon deciduousness is a broad categorization that captures many physiological differences, including for evergreen species, generally slower growth rates, less nutrient rich leaves and litterfall, abundance in cooler more nutrient poor environments, and shorter lifespans (Chapin et al., 2002). Deciduousness was important for models predicting survival responses to N deposition and growth responses to S deposition. Specifically, evergreen species’ survival response to N deposition were more vulnerable to N deposition overall, with a higher proportion of decreasing and unimodal relationships compared with deciduous species. This observation agrees with earlier work, where survival of an evergreen species (*Pinus resinosa*) in a pine plantation decreased in Harvard Forest and the survival of some deciduous trees decreased and others did not (Magill et al., 2004). However, there was a mix of vulnerable and resilient species in both broad groups. Evergreen species may be overall more vulnerable due to their increased susceptibility to N-induced acidification (Aber et al., 1998; Magill et al., 2000, 2004) or additional growth when N is available becomes susceptible to stressors under less favorable environmental conditions (McNulty and Boggs, 2010), though deciduous species also have shown sensitivity (May et al., 2005).

#### 4.2.5 Mycorrhizal association

Mycorrhizal association and soil pH were influential in predicting survival response to S deposition. Interestingly, trees associated with AM-fungi were more likely to exhibit flat or sigmoidal responses across the soil pH gradient, while trees associated with EcM-fungi were predicted to exhibit more vulnerable responses under lower soil pH conditions (pH ⪅ 5.5) and less vulnerable as soil pH increased; however, we report this result with caution as our model has high uncertainty above a pH of 7. Mycorrhizal associations did not emerge for relationships between N deposition and growth in the selected model, which was unexpected based on associations found in earlier work (Averill et al., 2018). They did emerge in the global (myco, *P* = 0.048) and the full hypothesis-driven models (N:myco, *P* = 0.06), with the expected associations; but, these models had a lower AIC than the selected model. These broad microbial groups, however, influence N cycling differently, and interact with other environmental factors, and thus a more nuanced approach to tease out the microbial signal may be warranted.

### 4.3 Limitations and uncertainties

While this analysis expands upon previous work to account for regional variation in tree species responses, there are limitations of this study. First, there was a large amount of unexplained variation in our models assessing the covariation between responses and climate, soil, and tree functional traits. This may result from the exclusion of additional factors that could be correlated with N and S or affect the responses to N and S include ozone (Fenn et al., 2020), CO_2_, stand aging, and pests among others. Our use of a spatial scale (USFS Divisions) precludes the integration of additional, potentially important variation in geology, landforms, soils, vegetation, climate, land use, wildlife, and hydrology in our models. The variation that was explained may be affected by confounding factors (e.g., correlations between N and S deposition, soil pH, and precipitation) therefore likely working in concert to drive variation in tree response. Second, recent research found that using the first and last measurement period for CONUS-wide analysis may introduce a negative bias for survival for some species (Clark et al., in press). It is unclear whether and to what degree this issue may be prevalent in our results given that we also analyzed species separately by division, which theoretically would reduce the bias given that most states do not have many remeasurements. Third, other methods for deriving growth and survival curves might permit more flexible responses, such as machine learning (Pavlovic et al., 2023). Fourth, it has been reported that space-for-time substitutions may not be effective to anticipate future responses to environmental change (Klesse et al., 2020; Perret et al., 2024). It is unclear how much this limitation affects our conclusions, given that our data are matched in space and across time; and it is unknown whether this incongruence, which is often reported for climate responses, also holds for other stressors like atmospheric deposition, suggesting that this remains an area in need of further study.

## 5 Conclusions

We described the regional variation in relationships between N and S deposition and the growth and survival of roughly 140 tree species across the U.S. To optimize management relevance, we grouped our tree species into populations based on Bailey’s Division-scale ecoregions and found there was substantial variation in tree species growth and survival responses across the U.S., with vulnerable species to atmospheric deposition in almost every region. There are many improvements to the previous analyses embodied here including the regionalization of assessed vulnerability, improvements in the characterization of responses to S deposition to be more ecologically realistic, and the inclusion of more factors to explain the variation found. Notwithstanding these, there remain many improvements to be made, including the inclusion of other co-variates that influence the tree responses (e.g., ozone, CO_2_, site fertility, base cation weathering, other soil chemical, and physical properties), the use of other systems for regionalization (e.g., Omernik), and extending these analyses to other forested regions of the U.S. (e.g., Alaska, Puerto Rico, and Hawaii).

Our analysis of covariates that correlate to the probability of tree responses indicated the interactions driving growth and survival responses to a pollutant were complex and varied, and there were likely more important factors driving variation that remain untested. First, soil pH was correlated with N-growth, and S-growth and S-survival in the context of interactions with other soil and tree functional trait factors. Second, contemporary N and S deposition considerably affected tree response to N and S deposition, but their specific impacts depend on the response. Third, tree functional traits (deciduousness and mycorrhizal association) are important as single effects and through interactions with deposition and soil pH in predicting species’ growth response to N deposition (deciduousness) and survival responses to S deposition (soil pH and N deposition × mycorrhizal association). Overall, this work helps better understand the regional variation in tree responses to N and S deposition in the U.S., and the factors that influence that vulnerability.

## Supplementary Material

Supplement1

Supplement2

Supplement3

## Data Availability

Publicly available datasets were analyzed in this study. This data can be found here: the data and R code that supports the findings of this study are available at the EPA Science Hub data repository (https://doi.org/10.23719/1529764). A description of all data files is available in Supplemental material 3.
