# Supplementary material for: Regional variation in growth and survival responses to atmospheric nitrogen and sulfur deposition for 140 tree species across the United States
Source: Front For Glob Change. Author manuscript; Available in PMC 2025 Nov 11. (PMC11864324; doi:10.3389/ffgc.2024.1426644)
Supplement: Supplement1 [file NIHMS2038564-supplement-Supplement3.pdf]

Supplementary Material 1

Title: Regional variation in growth and survival responses to atmospheric nitrogen and sulfur deposition for 140 tree species across the U.S.

Authors: Rebecca M. Dalton[1], Jesse N. Miller[2], Tara Greaver[1], Robert D. Sabo[2], Kemen G. Austin[3], Jennifer N. Phelan[3], R. Quinn Thomas[4,5], Christopher M. Clark[2]

Institutional affiliations:

[1] U.S. Environmental Protection Agency, Office of Research and Development, Center for Public Health and Environmental Assessment, Research Triangle Park, Durham, NC, United States of America

[2] U.S. Environmental Protection Agency, Office of Research and Development, Center for Public Health and Environmental Assessment, Washington, DC, United States of America

[3]Research Triangle Institute (RTI) International, Research Triangle Park, NC, United States of America

[4]Department of Forest Resources and Environmental Conservation, Virginia Tech, Cheatham Hall, Blacksburg, VA, United States of America

[5] Department of Biological Sciences, Virginia Tech, Derring Hall, Blacksburg, VA, United States of America

List of items in Supplementary Material 1:

- **Figure S1**. Map of USFS Divisions
- **Figure S2**. Tree variation in growth and survival responses across multiple USFS Divisions
- **Figure S3.** Distribution of tree species' responses across the CONUS
- **Figure S4**. Predicted tree growth to S deposition across single covariates
- **Figure S5**. Predicted tree survival to S deposition across single covariates

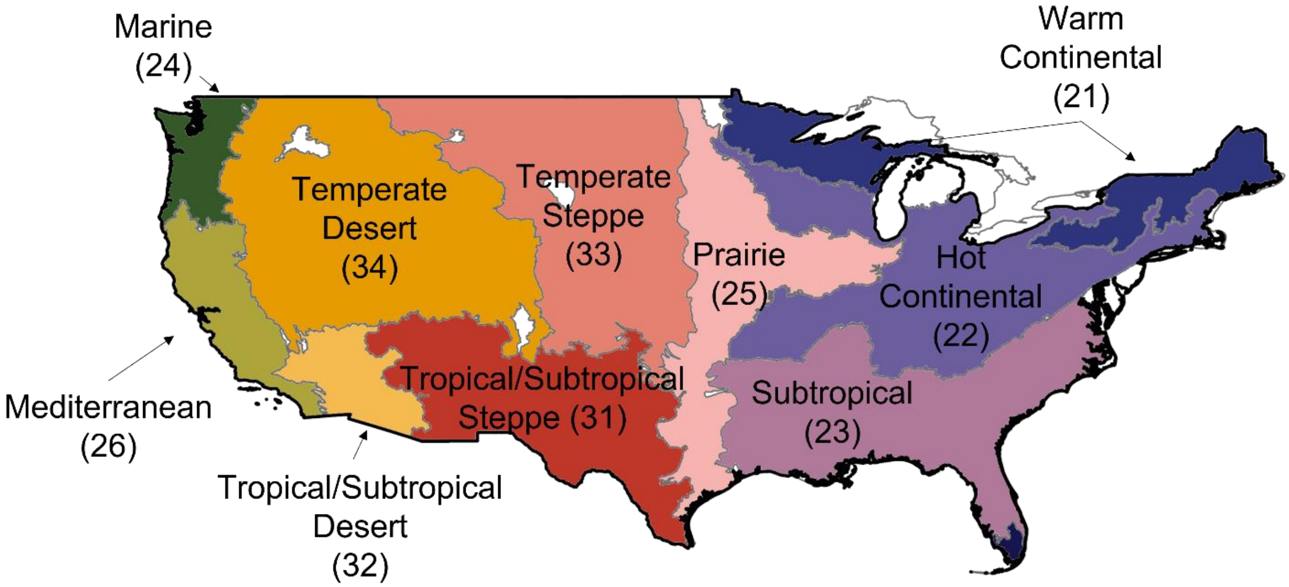

Figure S1. Map of the United States Forest Service Divisions in the conterminous United States.

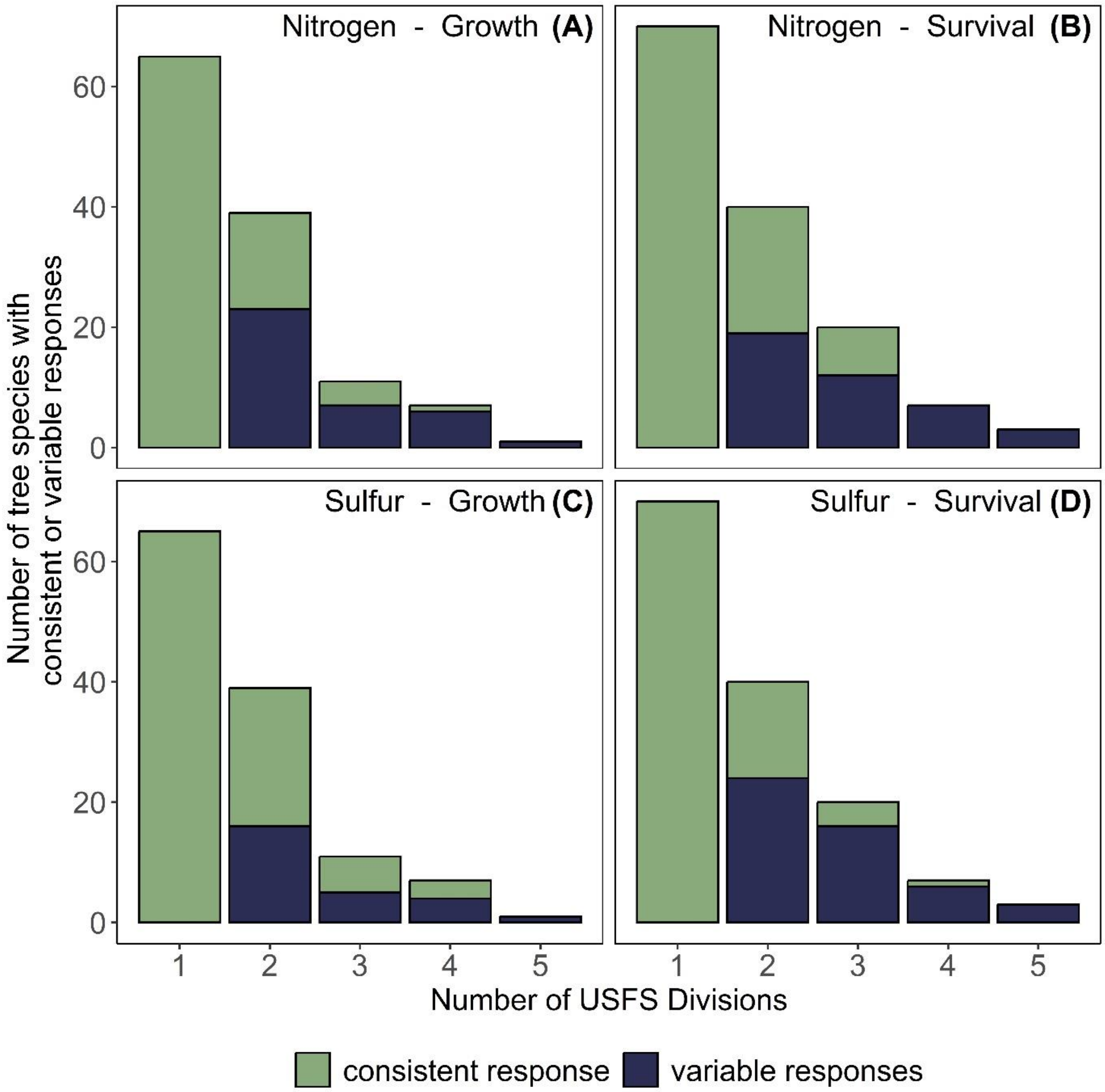

Figure S2. Most tree species show variable growth and survival responses across USFS Divisions. The number of tree species with consistent (green) and variable (purple) growth or survival responses to Nitrogen and Sulfur deposition across USFS Divisions. Consistent responses indicate a uniform growth/survival response to deposition (e.g., flat in Division 21 and Division 32) while variable responses include tree species with multiple responses (e.g., flat in Division 21 and decrease in Division 32.

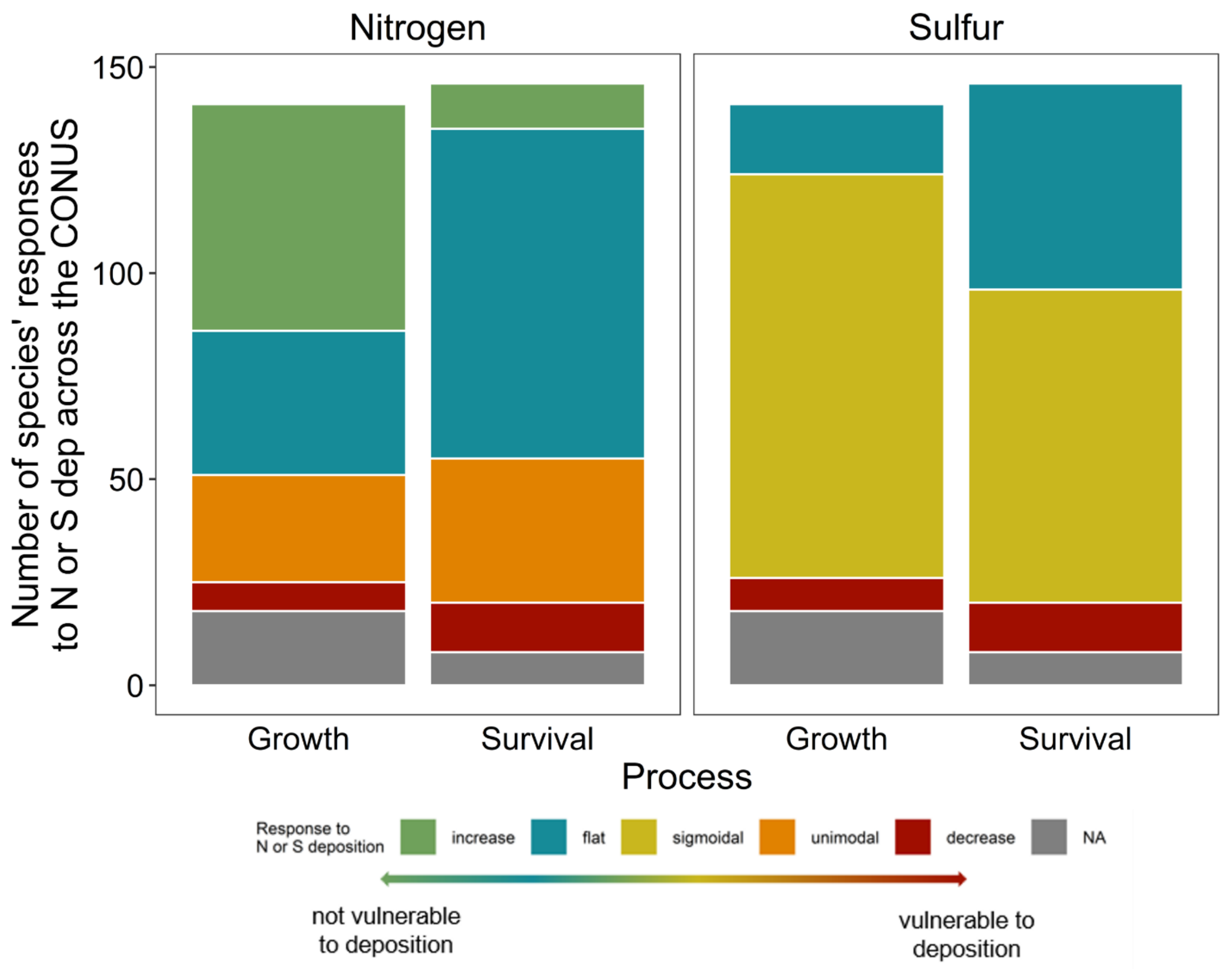

Fig. S3. Distribution of responses across conterminous United States (CONUS). The number of tree species across the conterminous United States (CONUS) with flat (blue), increasing (green), decreasing (red), unimodal (orange, N deposition only), sigmoidal (yellow, S deposition only) aboveground tree growth (kg C tree^{-1} year^{-1}) and survival (P(s) 10 yr^{-1}) responses to increasing N deposition (kg N ha^{-1} yr^{-1}) and S deposition (kg S ha^{-1} yr^{-1}). Note that we included a total of 141 species for growth relationships, and 145 species for survival relationships across the CONUS. Not applicable (gray) indicates that N deposition and S deposition were highly correlated and tree growth and survival responses to N or S alone were unable to be calculated.

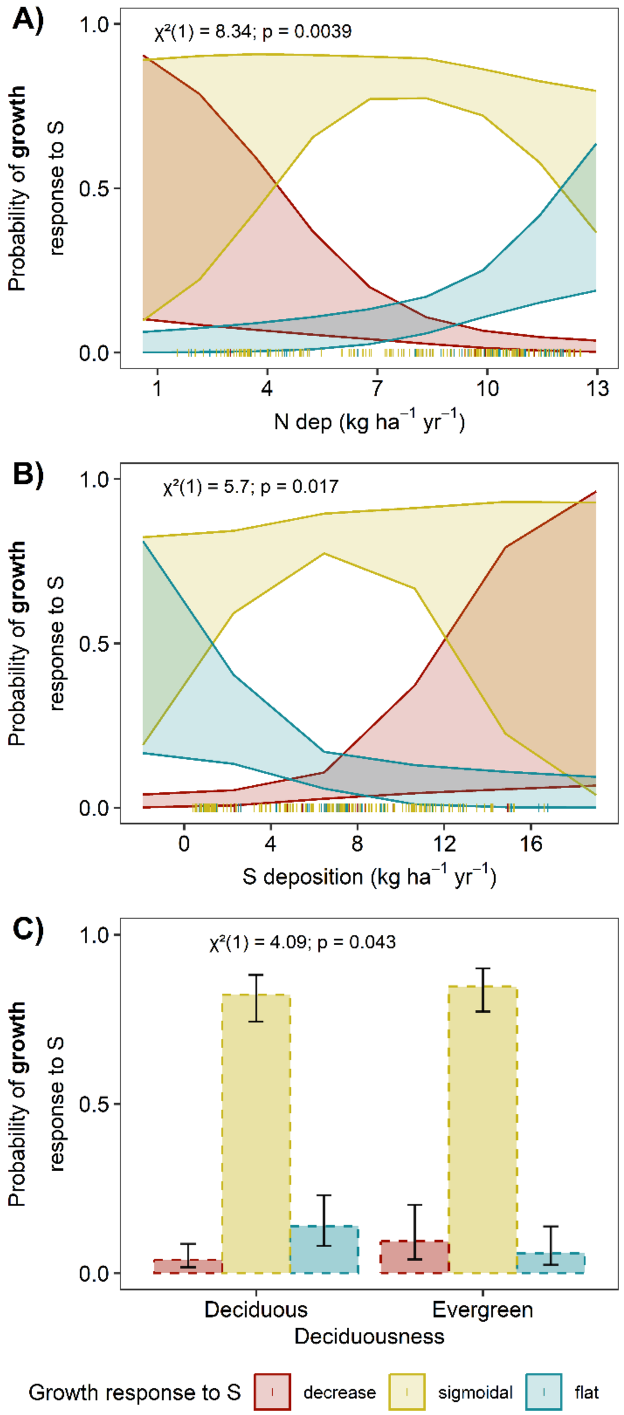

Figure S4. Predicted tree growth responses to S deposition. Relationship between how the distribution of S-growth responses are predicted to change along significant main effects of atmospheric deposition. For S-growth, A) N deposition, B) S deposition, and C) deciduousness are shown. The y-axes are the predicted probability of a flat (orange), unimodal (blue), or decreasing (red) S-growth relationship as the environmental drivers changes. Shaded bands represent the 95% confidence intervals. Chi-square (χ^2), degrees of freedom, and p-values are reported for each covariate in the upper left corner.

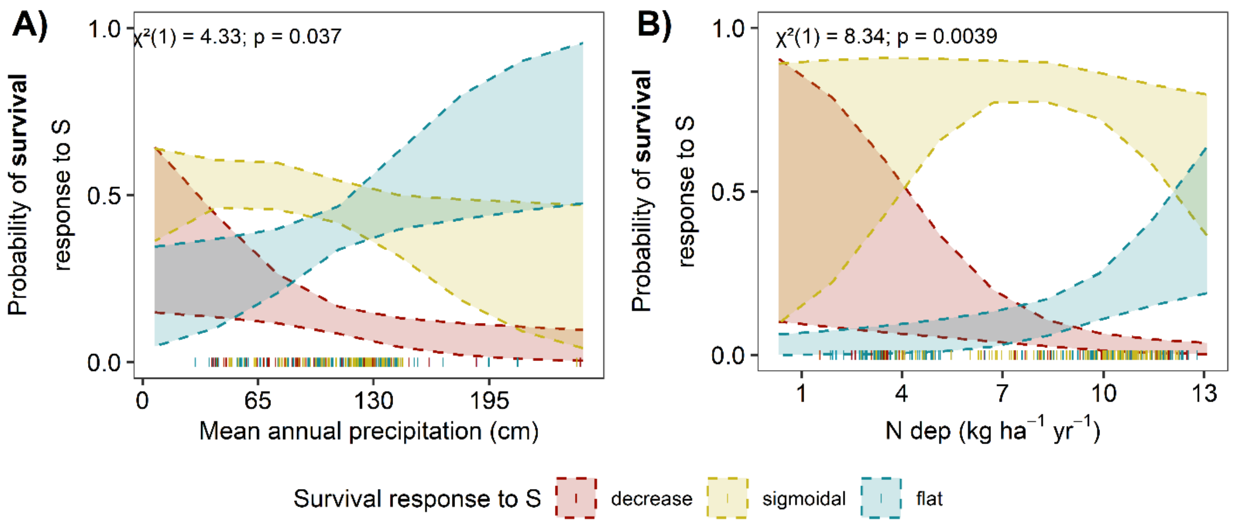

Figure S5. Predicted tree survival responses to S deposition. Relationship between how the distribution of S-survival responses are predicted to change along significant main effects of environmental drivers. For S-survival, A) mean annual precipitation (cm) and B) S deposition are shown. The y-axes are the predicted probability of a flat (orange), unimodal (blue), or decreasing (red) S-growth relationship as the environmental drivers changes. Shaded bands represent the 95% confidence intervals. Chi-square (χ^2), degrees of freedom, and p-values are reported for each covariate in the upper left corner.
