# Supplementary material for: Regional variation in growth and survival responses to atmospheric nitrogen and sulfur deposition for 140 tree species across the United States
Source: Front For Glob Change. Author manuscript; Available in PMC 2025 Nov 11. (PMC11864324; doi:10.3389/ffgc.2024.1426644)
Supplement: Supplement3 [file NIHMS2038564-supplement-Supplement2.pdf]

Supplementary Material 3
Description of datasets available in EPA's ScienceHub Catalog

Title: Regional variation in growth and survival responses to atmospheric nitrogen and sulfur deposition for 140 tree species across the United States

Authors:

Rebecca M. Dalton[1], Jesse N. Miller[2], Tara Greaver[1], Robert D. Sabo[2], Kemen G. Austin[3], Jennifer N. Phelan[3], R. Quinn Thomas[4,5], Christopher M. Clark[2]

Institutional affiliations:

[1] U.S. Environmental Protection Agency, Office of Research and Development, Center for Public Health and Environmental Assessment, Research Triangle Park, Durham, NC, United States of America

[2] U.S. Environmental Protection Agency, Office of Research and Development, Center for Public Health and Environmental Assessment, Washington, DC, United States of America

[3]Research Triangle Institute (RTI) International, Research Triangle Park, NC, United States of America

[4]Department of Forest Resources and Environmental Conservation, Virginia Tech, Cheatham Hall, Blacksburg, VA, United States of America

[5] Department of Biological Sciences, Virginia Tech, Derring Hall, Blacksburg, VA, United States of America

Link to EPA's ScienceHub directory for data related to this manuscript:
https://doi.org/10.23719/1529764

Data files available:

1. **Tree_CLA1_RCode-2024-FFCG.R**: R code for compiling datasets, running ordinal analyses, and creating visualizations. This code was last updated by RMD on 31 May 2023.

2. **TreeCLA1_combined_df_FFGC_2024.csv:** Dataset containing selected models characterizing tree growth and survival responses to N and S deposition across the CONUS and USFS Divisions along with associated climate, edaphic, deposition, and tree functional trait covariates. This dataset is compiled in "**Tree_CLA1_RCode-2024- FFGC.R"** and has an additional tab containing metadata titled "README."

3. **TreeCLA1_finalmodeloutputs_2021.xlsx**: Final model outputs from RTI International. This file includes all model combinations for determining tree species survival and growth responses for 145 tree species across 11 USFS Divisions. See "README" tab for a description of tabs, and "GrowthDIVmeta", "GrowthCONUSmeta", "SurvivalDIVmeta," and "SurvivalCONUSmeta" for descriptions of all parameters.

4. **Jo2019_data_tree_sp_myc_type.xlsx:** mycorrhizal association data from Jo et al. (2019) received file via email to C. Clark.

5. **DIVISION_fia_forestdivision_temp_precip_pH_with species_ALL SPP.csv** and **CONUS_fia_forestdivision_temp_precip_pH_with species_ALL SPP.csv.**: We extracted the 30-year mean annual temperature (MAT) and precipitation (MAP) (1991 - 2020) at the USDS FIA plot level using the PRISM climate normals (https://prism.oregonstate.edu/normals/) (Daly et al. 2008) and deposition data from the National Atmospheric Deposition Program from 2000 – 2012 (Schwede and Lear 2014). Likewise, we extracted soil pH estimates at the FIA plot level from the Gridded National Soil Survey Geographic Database (https://www.nrcs.usda.gov/resources/data-and-reports/gridded-national-soil-survey-geographic-database-gnatsgo). We determined mean climatic, soil pH, and deposition conditions for a species, within a forest Division and across the U.S., by averaging all the plot-level temperature, precipitation, soil pH, and N and S deposition observations where a specific species was observed.

6. **Horn2018_SpeciesList.csv** : Tree deciduousness (E or D) and the number of species in Horn et al. (2018). Adapted from Supplemental File 1 in Horn et al., 2018 on 25 Jan 2022.

7. **Kattge2020_decid.txt**: Vegetative phenology (or deciduousness) for tree species in our analysis (TraitID = 37) from the TRY plant trait database (Kattge et al. 2011).

8. **EcoMapDivisions_centroids_Table.xlsx:** Data file which includes centroid points for divisions. Created by J. Miller.

9. **Map_Divisions.png**: outline of division map from USFS

10. **Potterwoodall2012_phylogenetic.txt**: Phylogenetic supertree file from Potter and Woodall (2012)

References:

Daly, C., M. Halbleib, J. I. Smith, W. P. Gibson, M. K. Doggett, G. H. Taylor, J. Curtis, and P. P. Pasteris. 2008. Physiographically sensitive mapping of climatological temperature and precipitation across the conterminous United States. International Journal of Climatology: a Journal of the Royal Meteorological Society **28**:2031-2064.

Horn, K. J., R. Q. Thomas, C. M. Clark, L. H. Pardo, M. E. Fenn, G. B. Lawrence, S. S. Perakis, E. A. H. Smithwick, D. Baldwin, S. Braun, A. Nordin, C. H. Perry, J. N. Phelan, P. G. Schaberg, S. B. St Clair, R. Warby, and S. Watmough. 2018. Growth and survival relationships of 71 tree species with nitrogen and sulfur deposition across the conterminous U.S. PLoS One **13**:e0205296.

Kattge, J., S. Diaz, S. Lavorel, I. C. Prentice, P. Leadley, G. Bönisch, E. Garnier, M. Westoby, P. B. Reich, and I. J. Wright. 2011. TRY–a global database of plant traits. Global change biology **17**:2905-2935.

Potter, K. M., and C. W. Woodall. 2012. Trends over time in tree and seedling phylogenetic diversity indicate regional differences in forest biodiversity change. Ecological applications **22**:517-531.

Schwede, D. B., and G. G. Lear. 2014. A novel hybrid approach for estimating total deposition in the United States. Atmospheric Environment **92**:207-220.
